# *ATP6AP1* deficiency causes an immunodeficiency with hepatopathy, cognitive impairment and abnormal protein glycosylation

**DOI:** 10.1038/ncomms11600

**Published:** 2016-05-27

**Authors:** Eric J. R. Jansen, Sharita Timal, Margret Ryan, Angel Ashikov, Monique van Scherpenzeel, Laurie A. Graham, Hanna Mandel, Alexander Hoischen, Theodore C. Iancu, Kimiyo Raymond, Gerry Steenbergen, Christian Gilissen, Karin Huijben, Nick H. M. van Bakel, Yusuke Maeda, Richard J. Rodenburg, Maciej Adamowicz, Ellen Crushell, Hans Koenen, Darius Adams, Julia Vodopiutz, Susanne Greber-Platzer, Thomas Müller, Gregor Dueckers, Eva Morava, Jolanta Sykut-Cegielska, Gerard J. M. Martens, Ron A. Wevers, Tim Niehues, Martijn A. Huynen, Joris A. Veltman, Tom H. Stevens, Dirk J. Lefeber

**Affiliations:** 1 Department of Molecular Animal Physiology, Donders Institute for Brain, Cognition and Behaviour, Centre for Neuroscience and Radboud Institute for Molecular Life Sciences, Faculty of Science, Radboud University, 6525 GA Nijmegen, The Netherlands; 2 Department of Neurology, Donders Institute for Brain, Cognition and Behaviour, Radboud University Medical Center, 6525 GA Nijmegen, The Netherlands; 3 Department of Laboratory Medicine, Translational Metabolic Laboratory, Radboud Institute for Molecular Life Sciences, Radboud University Medical Center, 6525 GA Nijmegen, The Netherlands; 4 Department of Chemistry and Biochemistry, Institute of Molecular Biology, University of Oregon, Eugene, Oregon 97403, USA; 5 Metabolic Unit, Rambam Health Care Center, Rappaport School of Medicine, Technion, 3109601 Haifa, Israel; 6 Department of Human Genetics, Radboud Institute for Molecular Life Sciences and Donders Centre for Neuroscience, Radboud University Medical Center, 6525 GA Nijmegen, The Netherlands; 7 The Milman-David Biomedical Research Unit, 24 Hazevi Avenue, 34355 Haifa, Israel; 8 Department of Laboratory Medicine and Pathology, Mayo College of Medicine, Rochester, Minnesota 55905, USA; 9 Research Institute for Microbial Diseases, Osaka University, Suita, Osaka 565-0871, Japan; 10 Department of Pediatrics, Nijmegen Centre for Mitochondrial Disorders (NCMD), Radboud university medical center, 6525 GA Nijmegen, The Netherlands; 11 Protein Laboratory, Children's Memorial Health Institute, 04730 Warsaw, Poland; 12 Temple Street Children's University Hospital, Temple Street, Dublin 1, DC01 YC67, Ireland; 13 Department of Laboratory Medicine, Medical Immunology, Radboud University Medical Center, 6525 GA Nijmegen, The Netherlands; 14 Personalized Genomic Medicine Pediatric Genetics and Metabolism Goryeb Children's Hospital, Morristown, New Jersey 07960, USA; 15 Department of Pediatrics and Adolescent Medicine, Medical University of Vienna, 1090 Vienna, Austria; 16 Department of Pediatrics I, Medical University of Innsbruck, 6020 Innsbruck, Austria; 17 HELIOS Kliniken Krefeld, Children's Hospital, Lutherplatz 40, 47805 Krefeld, Germany; 18 Department of Pediatrics, Tulane University Medical School, New Orleans, Los Angeles 70112, USA; 19 Department of Pediatrics, University Medical School of Leuven, 3000 Leuven, Belgium; 20 Department of Pediatrics, Radboudumc, 6525GA, Nijmegen, The Netherlands; 21 Screening Department, Institute of Mother and Child, 01-211 Warsaw, Poland; 22 Centre for Molecular and Biomolecular Informatics, Radboud Institute for Molecular Life Sciences, Radboud University Medical Center, 6525GA Nijmegen, The Netherlands; 23 Department of Clinical Genetics, Maastricht University Medical Centre, 6229HX Maastricht, The Netherlands

## Abstract

The V-ATPase is the main regulator of intra-organellar acidification. Assembly of this complex has extensively been studied in yeast, while limited knowledge exists for man. We identified 11 male patients with hemizygous missense mutations in *ATP6AP1*, encoding accessory protein Ac45 of the V-ATPase. Homology detection at the level of sequence profiles indicated Ac45 as the long-sought human homologue of yeast V-ATPase assembly factor Voa1. Processed wild-type Ac45, but not its disease mutants, restored V-ATPase-dependent growth in Voa1 mutant yeast. Patients display an immunodeficiency phenotype associated with hypogammaglobulinemia, hepatopathy and a spectrum of neurocognitive abnormalities. Ac45 in human brain is present as the common, processed ∼40-kDa form, while liver shows a 62-kDa intact protein, and B-cells a 50-kDa isoform. Our work unmasks Ac45 as the functional ortholog of yeast V-ATPase assembly factor Voa1 and reveals a novel link of tissue-specific V-ATPase assembly with immunoglobulin production and cognitive function.

## Abstract (web summary)

Here, Dirk Lefeber and colleagues identify functional mutations in ATP6AP1 encoding Ac45. The authors show that Ac45 is the functional ortholog of yeast V-ATPase assembly factor Voa1 and provide evidence for tissue-specific Ac45 processing, associated with the clinical phenotype of immunodeficiency, hepatopathy, and neurocognitive abnormalities.

## Graphical abstract

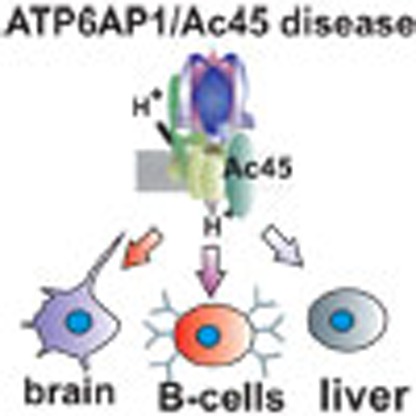

The vacuolar H^+^-ATPase (V-ATPase) is a ubiquitously expressed protein complex, required for luminal acidification of secretory vesicles to acidify the extracellular milieu, compartments of the endocytic pathway including lysosomes, and of the Golgi apparatus^1^,^2^. The V-ATPase consists of two multi-protein domains, V_1_ and V_0_. The peripheral V_1_ domain comprises eight subunits (A–H), is localized in the cytoplasm and hydrolyses ATP. The V_0_ domain is embedded in the organelle membrane, consists of five subunits (a, d, e, c and c″) and harbours the rotary mechanism for proton translocation^2^. Human disease mutations in V-ATPase core subunits result in distinct clinical syndromes. In 1999, renal tubular acidosis with deafness was the first phenotype linked to the V-ATPase with mutations in the kidney-specific isoforms *ATP6V1B1* (MIM 267300) or *ATP6V0A4* (MIM 602722) (refs 3, 4, 5). In 2000, osteopetrosis (MIM 259700) was linked to the V-ATPase by identification of mutations in *TCIRG1*, encoding the osteoclast-specific a3 subunit^6^,^7^. In 2008, mutations were found in *ATP6V0A2* in a subgroup of cutis laxa syndromes with abnormal protein glycosylation, autosomal recessive cutis laxa type II (MIM 219200&278250). *ATP6V0A2* encodes the a2 subunit, which localizes the V-ATPase complex to the Golgi apparatus^8^.

In addition to the core V-ATPase subunits, two accessory proteins are known in vertebrates, that is, ATP6AP1 (also known as Ac45) and ATP6AP2 (also known as (Pro-) renin receptor)^9^. In vertebrates, Ac45 is ubiquitously expressed with the highest levels in neuronal and (neuro-) endocrine cells and osteoclasts^10^,^11^,^12^,^13^. This accessory subunit of the proton pump guides the V-ATPase into specialized subcellular compartments such as neuroendocrine regulated secretory vesicles^14^,^15^ or the ruffled border of the osteoclast^10^,^16^,^17^ thereby regulating its activity. Moreover, the Ac45 protein is involved in membrane trafficking and Ca^2+^-dependent membrane fusion^18^. V-ATPase assembly has been extensively studied in yeast, where Vma21, Vma12 and Vma22 cooperate in the assembly of the V_0_ domain in the endoplasmic reticulum (ER) membrane^19^,^20^,^21^,^22^,^23^. Additionally, yeast Voa1 has been established as an ER-localized V_0_-assembly factor in 2008. However, no human orthologue has been identified so far^24^. In human, V-ATPase assembly is hardly studied, and no yeast orthologue of human Ac45 has thus far been identified^9^,^25^,^26^,^27^.

In this study, we describe a novel ATP6AP1-linked immunodeficiency and identified disease mutations in *ATP6AP1* in 11 male patients with abnormal protein glycosylation. Yeast V-ATPase assembly factor Voa1 was predicted to be homologous to Ac45, which was confirmed by functional complementation of Voa1 mutant yeast with the processed C-terminal domain of Ac45. Identification of different Ac45 protein isoforms in human brain, liver and B cells indicated the presence of tissue-specific regulation of organelle acidification.

## Results

### Identification of mutations in X-linked *ATP6AP1*

**Figure 1:**
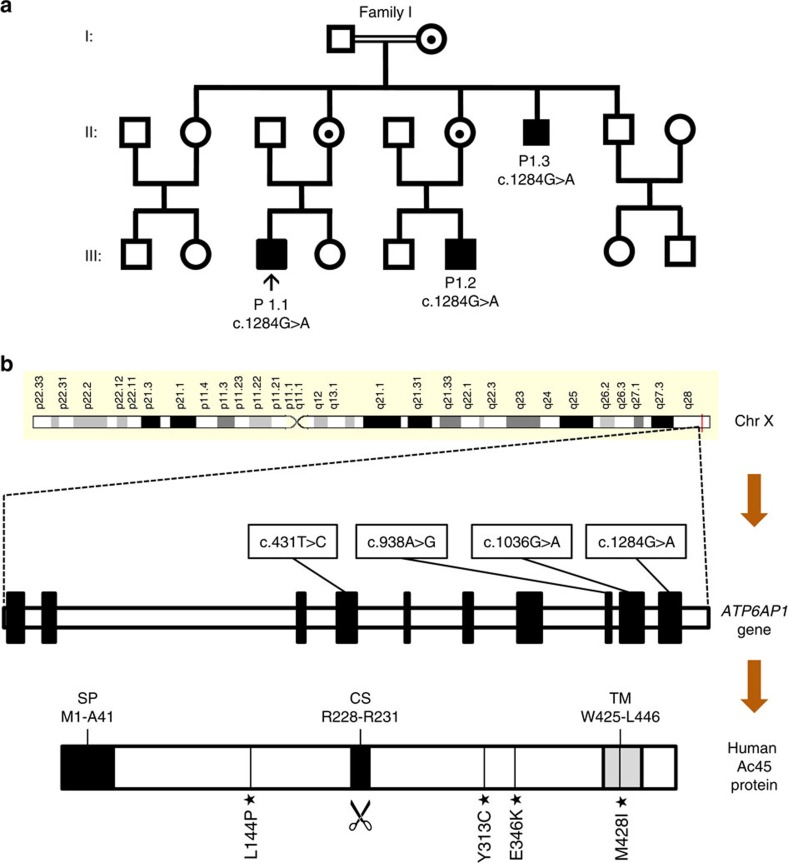
Overview of identified *ATP6AP1* mutations. (**a**) Pedigree of index family 1. Black arrow (↑) indicates the index patient P1.1 with the mutation c.1284G>A (p.Met428Ile). (**b**) *ATP6AP1 g*ene, located on chromosome Xq28, and its gene structure consisting of 10 exons. All mutations identified in the six families are indicated in boxes. Domain structure of Ac45 as published on uniprot.org for human Ac45 (http://www.uniprot.org/uniprot/Q15904; Entry version 146, 07 January 2015). CS, furin proteolytic cleavage site^30^; SP, signal peptide; TM, transmembrane region. Stars (★) indicate the location of the mutations at the protein level. See also Supplementary Fig. 1.

In our cohort of unsolved patients with deficient glycosylation of proteins, we performed exome sequencing to identify the causative gene defect. Exome sequencing of a male patient (individual 1.1, Table 1) was performed as previously described^28^. After filtering out poor-quality variants as well as common and synonymous variants (see Methods section), 131 rare missense variants were selected. Based on a recessive inheritance model, two candidate genes remained (Supplementary Table 1): X-linked *ATP6AP1* with hemizygous variant and *KPRP* on Chr1 with compound heterozygous variants. Of these variants, the c.1284G>A variant in *ATP6AP1* on chrXq28 showed the highest level of conservation (PhyloP 46-way, 5.1) and was predicted to be pathogenic by Sift, Polyphen-2 and MutationTaster. Moreover, no potentially pathogenic variants were identified in *KPRP* in the WES data of patients 2.1 and 6.1. *ATP6AP1* encodes the accessory subunit Ac45 of the V-ATPase complex^13^, the proton pump that has been linked with abnormal glycan processing in the Golgi via mutations in its core subunit *ATP6V0A2* (ref. 8). Sanger sequencing confirmed the hemizygous missense mutation (c.1284G>A, p.M428I) in the patient as well as in two affected male family members that became known during the sequencing process (Fig. 1). All maternal alleles showed heterozygosity and healthy males were hemizygous wild type (Supplementary Fig. 1A), confirming complete segregation of the c.1284G>A mutation with disease in agreement with X-linked inheritance. Exome and Sanger sequencing of *ATP6AP1* in a cohort of unsolved male patients with abnormal protein glycosylation revealed additional mutations in eight patients from five families (Table 1, Supplementary Fig. 1B). Patient 2.1 showed a c.431T>C (p.L144P) missense mutation, heterozygous in the mother and absent from the father and a healthy sister. An additional hemizygous missense mutation (c.1036G>A, p.E346K) was identified by Sanger sequencing in three non-related male sib pairs (families 3–5). Fathers carried wild-type alleles and mothers were heterozygous for the c.1036G>A variant, in agreement with X-linked inheritance. Exome sequencing of patient 6.1 revealed a c. 938A>G (p.Y313C) missense mutation.

All four missense mutations (L144P, Y313C, E346K and M428I) affect amino acids that are highly conserved down to fruitfly, tetraodon and frog (Supplementary Fig. 1C). Ac45 homologues in more distantly related species could not readily be retrieved by use of standard BLAST searches. L144P is located in the N-terminal domain, while Y313C, E346K and M428I are located in the processed C-terminal domain of Ac45 (Fig. 1b).

### Clinical phenotype of Ac45 deficiency

The dominating clinical symptoms displayed by the present cohort of patients with various *ATP6AP1* mutations (Table 1) include hepatopathy and immune abnormalities. Recurrent bacterial infections were associated with hypogammaglobulinemia, ranging from plantar abscesses and gastrointestinal infections in family 1 to multiple episodes of childhood pneumonia and purulent otitis media in families 2–6. Several patients were successfully treated with intravenous immunoglobulins. Of note, some of the patients responded very poorly to childhood vaccinations. Hepatopathy ranged from mild hypertransaminasemia to cirrhosis and end-stage liver failure. In addition, gastric problems were noted in the majority of patients and laboratory abnormalities included leukopenia, slightly elevated serum transaminases, low serum copper and ceruloplasmin, and high alkaline phosphatase. The two brothers from family 4 displayed high-normal levels of IgD+/CD27− naïve B cells and lowered levels of IgD+/CD27+ intermediate and switched memory B cells, suggesting a problem in B-cell differentiation.

Patients with the p.E346K substitution (families 3–5) showed in addition splenomegaly, abnormal hepatic histology and neurological symptoms such as epilepsy, mild intellectual disability and behaviour abnormalities. Muscle weakness with mildly elevated serum creatine kinase (CK) was demonstrated in a few patients, including patient 6.1. Of note, the presence of the p.E346K mutation predicted a more severe phenotype within the ATP6AP1 disease spectrum, which is in accordance with the early death of two patients (3.2 and 5.1) due to liver failure. Patients from families 1, 2 and 6 display a milder disease course and are currently doing well without cognitive impairment.

The index family (substitution p.M428I) presented with a milder disease course (oldest patient 34 years of age). All three patients in this kindred presented with sensorineural hearing loss to various extents and hyperopia. The grandmother also presented deafness at older age. The vision abnormality was documented also in patients 3.1 and 4.1 (E346K substitution). Female carriers did not show any obvious clinical symptoms. In the total cohort of patients, only minor clinical symptoms were observed that overlap with symptoms from known genetic defects in the V-ATPase, including deafness (*ATP6V1B1* and *ATP6V0A4*) and muscle weakness, as reported for X-linked Myopathy with Excessive Autophagy (XMEA, MIM 310440) due to mutations in V-ATPase assembly factor *VMA21* (ref. 29). When performed, radiography showed no signs of osteopetrosis as observed in ATP6V0A3 deficiency. There was no apparent renal phenotype with no signs of metabolic acidosis. Hypokalemia was reported for patients 3.1 and 5.1. In general, no signs for renal tubulopathy as observed in ATP6V0A4 deficiency were seen.

### Liver biopsy findings

**Figure 2:**
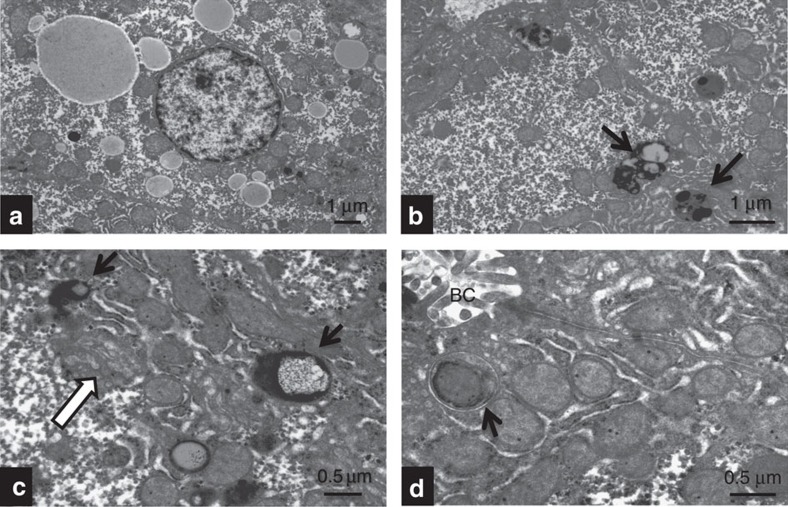
Ultrastructural studies of a liver biopsy of patient 3.1. (**a**) A hepatocyte is surrounded by fat globules of variable size having the typical aspect of triglycerides, × 4,000. (**b**) Hepatocyte showing relative translucency due to proliferated SER. Arrows point to lipofuscin bodies (lysosomes), × 6,000. (**c**) Higher magnification showing an atypical lipofuscin body with a central reticulate content (black arrow). White arrow: section through a Golgi apparatus, × 10,000. (**d**) A mitochondrion is engulfed in an autophagosome (arrow). BC, Bile canaliculus, × 12,000.

Liver biopsy was performed in six patients and was (near)-normal for patients 1.3 and 2 (with the substitutions p.M428I and p.L144P, respectively), but revealed steatosis, fibrosis and even micronodular cirrhosis in patients with the p.E346K mutation (Table 1, Supplementary Fig. 2). Electron microscopy was performed in a liver tissue specimen of patient 3.1 (Fig. 2). Hepatocytes showed variable translucency due to the presence of noticeable proliferating smooth endoplasmic reticulum, and of numerous alpha-glycogen monoparticles, accumulated within the cell centre. At the periphery, mitochondria were disposed along the plasma membrane (in a mode similar to that observed in glycogen storage diseases). Mitochondria were of usual size but frequently showed cristolysis and occasional absence of cristae. The dense-matrical bodies were preserved. No intra-mitochondrial crystals were noticed. Within hepatocytes, fat globules with the typical shape and size of triglycerides were seen, in variable amounts (Fig. 2a, Supplementary Fig. 2). Their sizes varied from small (1.5–6.0 nm) to medium (8.0–20 nm) and rarely large (21–40 nm). Lysosomes (single limit membrane) were identified as typical (Fig. 2b, arrows) and atypical lipofuscin bodies. The latter showed a central electron-lucent accumulation with a reticular network (Fig. 2c, arrows). Mitochondria were seen occasionally within phagosomes (autophagocytosis, Fig. 2d, arrow). No dilated or enlarged Golgi apparatus was detected in hepatocytes.

### Ac45 deficiency alters Golgi processing of protein glycans

**Figure 3:**
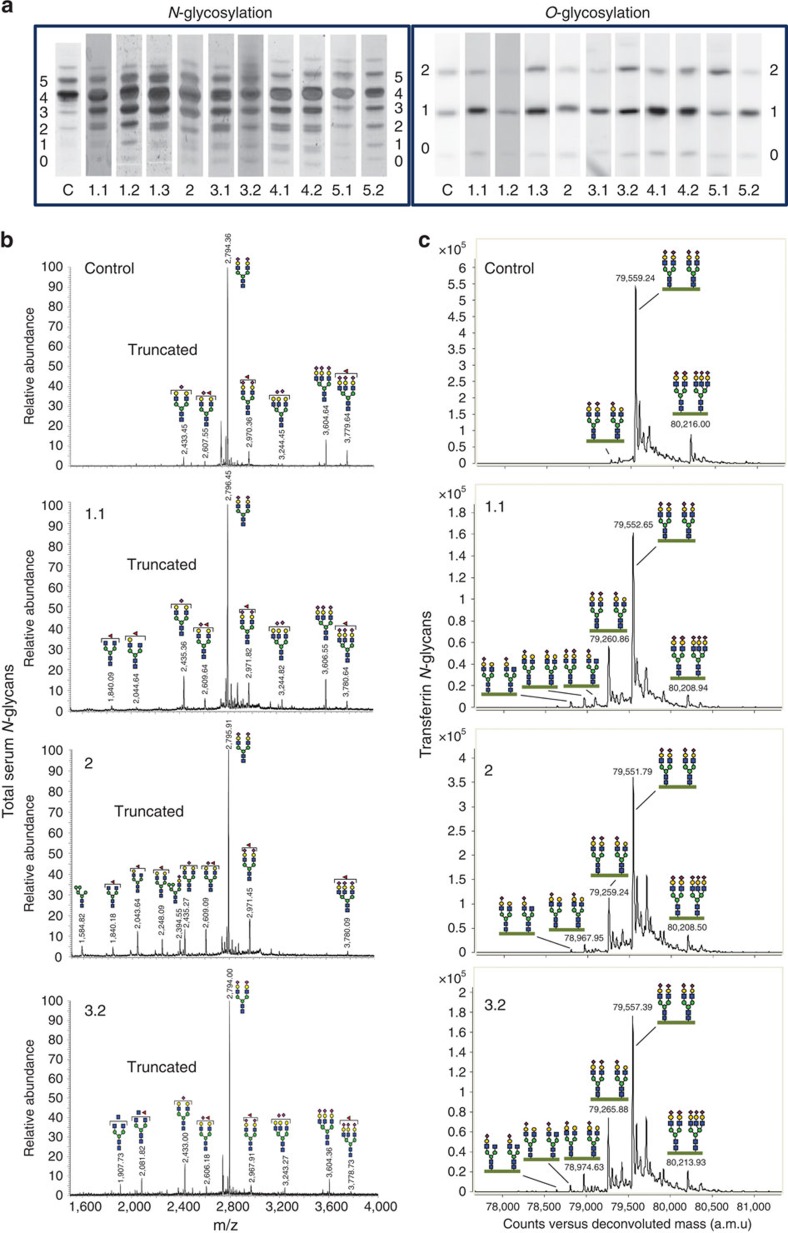
Glycosylation studies. (**a**) Routine screening for *N*-glycosylation by isofocusing of serum transferrin and for mucin type *O*-glycosylation by isofocusing of apolipoprotein CIII. The numbers on the *y* axis mark the number of sialic acids, each column shows the profile of the respective control (C) or patient. (**b**) Analysis of total serum protein *N*-glycans by MALDI mass spectrometry. (**c**) Analysis of intact serum transferrin by nanoLC-chip-QTOF mass spectrometry. Glycans are synthesized from individual monosaccharide building blocks: a purple diamond represents one sialic acid, a yellow circle one galactose, a green circle one mannose, a blue square one *N*-acetylglucosamine and a red triangle one fucose. See also Supplementary Fig. 3 and Supplementary Tables 2 and 3.

Analysis of protein *N*-glycosylation in serum showed abnormal profiles of transferrin in all studied patients (Fig. 3a, Supplementary Table 3). In addition, mucin type *O*-glycosylation of serum apolipoprotein CIII was abnormal in most patients, showing decreased sialylation. This combination of abnormal profiles is comparable with other defects of Golgi homoeostasis, such as ATP6V0A2-CDG (ref. 8). In patients 3.2 and 5.1 normal mucin type *O*-glycosylation was observed with even increased sialylation in patient 5.1. This might complicate recognition of Ac45-deficient patients as a genetically determined glycosylation disorder, since highly similar profiles are observed in patients with non-specific liver disease. Considerable variation in glycosylation abnormalities was observed in some of the patients tested (3.1 and 3.2). Mass spectrometry of total serum *N*-glycans revealed minor accumulations of truncated glycans (Fig. 3b) and no overlapping glycan signature could be regarded as specific for Ac45 deficiency. Mass spectrometric analysis of isolated transferrin revealed a clear accumulation of similar types of truncated glycans lacking galactose and sialic acid in all patients (Fig. 3c, Supplementary Fig. 3).

### Differential processing of Ac45 in liver, brain and B cells

**Figure 4:**
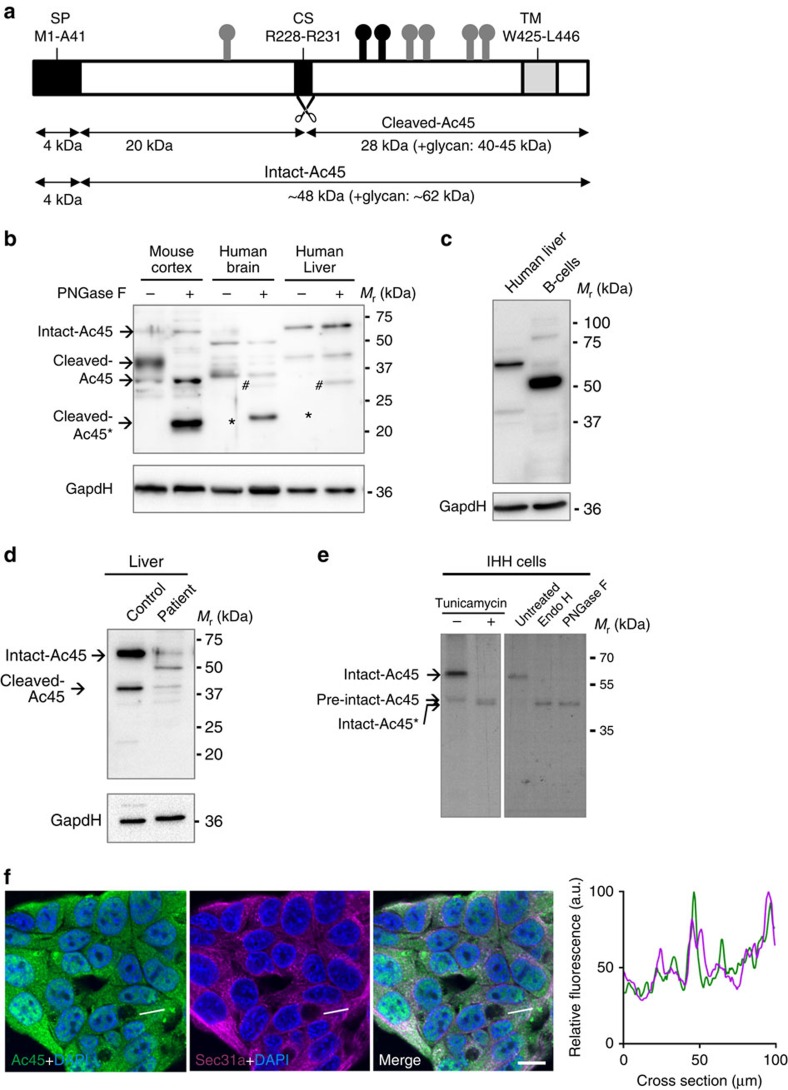
Differential expression of the Ac45 protein in human brain, liver and B cells. (**a**) Schematic representation of the human Ac45 protein. CS, furin proteolytic cleavage site; SP, signal peptide; TM, transmembrane domain; 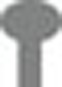 represent predicted *N*-glycan structures, whereas the structures shown in black (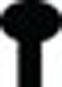) are the experimentally confirmed glycans^62^. (**b**) Western blot analysis of Ac45 in mouse cortex and in human brain and liver. Asterisk (*) indicates the deglycosylated form of cleaved-Ac45. Hash tags (#) indicate non-specific antibody reaction with PNGaseF present in the samples. (**c**) Western blot analysis of Ac45 in primary B cells from healthy controls in comparison with human liver. One of the two representative analyses is shown. (**d**) Western blot of Ac45 in liver tissue homogenates of control and patient 4.2. GapdH was used as loading control. (**e**) Analysis of newly synthesized Ac45 in immortalized human hepatocytes (IHH). Cells were transfected with Ac45 construct, pulsed for a 30-min period with ^35^S, and Ac45 was immunoprecipitated and analysed by SDS–PAGE. Cells were treated with or without tunicamycin during the 30-min pulse (left panel). Immunoprecipitated Ac45 protein was treated with or without Endo H or PNGaseF (right panel). Note during the 30-min pulse period, the presence of a minor portion of newly synthesized pre-intact-Ac45 protein is still in its unglycosylated proform and containing the signal peptide for translocation over the ER membrane. (**f**) IHH cells were stained with anti-Ac45 antibody (green) and antibodies against various organelle markers (magenta). Nuclear staining is shown in blue (DAPI). Co-localization is indicated by a white colour in the merged channel. The graph shows the fluorescent intensity profile along the cross-section indicated. Scale bar represents 10 μm. Staining for Sec31 is shown as example, other organelle markers are shown in Supplementary Fig. 5.

We studied *ATP6AP1* expression in human fetal and adult tissues. Both in fetal (data not shown) and adult tissues, the highest Ac45 mRNA expression was found in brain and the lowest expression level in liver and duodenum (Supplementary Fig. 4). To study Ac45 expression at the protein level, we performed western blot analysis of mouse cortex, human brain and liver, and human B cells using an Ac45 antibody directed to the C-terminal half of mouse Ac45. Ac45 is synthesized as a 62-kDa precursor protein (intact-Ac45) that in neuronal and neuroendocrine cells is subsequently processed to its ∼40-kDa cleaved form (cleaved-Ac45 (refs 12, 13, 15, 30), Fig. 4a). In mouse and human brain, most Ac45 protein was present in its cleaved ∼40 kDa form, with human Ac45 migrating slightly faster than its mouse counterpart (Fig. 4b, lanes 1 and 3). Furthermore, and in-line with earlier studies in *Xenopus* neuroendocrine cells^31^, these proteins were *N*-glycosylated as shown by their sensitivity towards endoglycosidase PNGaseF (Fig. 4b, lanes 2 and 4). These results are in agreement with the slightly lower molecular mass of human Ac45 as compared with mouse Ac45 and the presence of one extra *N*-glycan on mouse Ac45. In addition, in human brain a thus far unknown 50-kDa form was observed. In human and mouse liver, considerable Ac45 protein expression was observed, predominantly as the 62-kDa intact proteoform. Under the conditions used, this band was insensitive to PNGaseF treatment (Fig. 4b, lanes 5 and 6). Western blot analysis of primary B-cell isolates as well as B-cell lines (data not shown) revealed Ac45 as an ∼50-kDa protein isoform (Fig. 4c). Analysis of Ac45 protein expression in patient liver biopsy material revealed a strong reduction in the expression of the ∼62- and ∼40-kDa Ac45 variants and an additional ∼50-kDa protein was observed (Fig. 4d).

Subsequently, we performed newly synthesized protein labelling with ^35^S methionine in immortalized human hepatocytes (IHH), after transfection with GFP (mock) and wild-type human Ac45 complementary DNA. Immunoprecipitation with the Ac45 antibody revealed a dominant ∼62-kDa newly synthesized endogenous as well as exogenous Ac45 form (Supplementary Fig. 5A). Tunicamycin treatment of the IHH cells as well as PNGaseF digestion after labelling and immunoprecipitation revealed an ∼50-kDa Ac45 form, confirming *N*-glycosylation of the 62-kDa Ac45 isoform. In addition, EndoH sensitivity indicates the presence of high mannose glycans (Fig. 4e). Transfection of IHH cells with clinically relevant mutant Ac45 constructs showed similar expression as wild type (Supplementary Fig. 5B).

The subcellular localization of Ac45 in hepatocytes was studied by immunostaining of IHH cells. Ac45 was found to be mainly localized to the ER, and ER-to-Golgi Intermediate Compartment (ERGIC), but not to the *trans*-Golgi network (TGN) or components of the endosomal system (Fig. 4f, Supplementary Fig. 6).

### Human Ac45 is orthologous to yeast Voa1 and Big1

**Figure 5:**
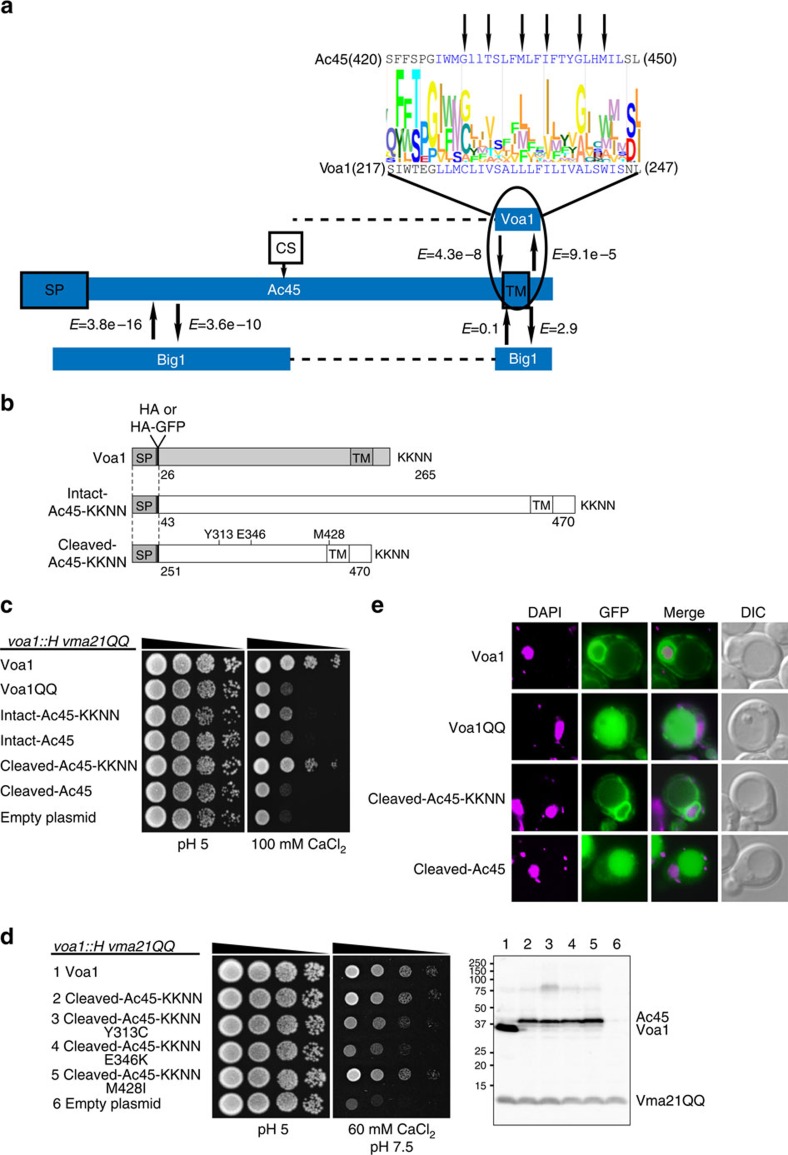
Identification of Voa1 as the yeast ortholog of human Ac45. (**a**) Overview of the regions of Ac45 that are homologous to the yeast proteins Voa1 and Big1 and an alignment of Ac45's and Voa1's C-terminal transmembrane helices (in blue, based on TMHMM^63^) and their flanking amino acids. Ac45 and Voa1 are separated by a sequence logo representation of this region among all the homologs that could be detected using JACKHMMER^64^. A pattern in which the level of sequence conservation in the transmembrane helix peaks every 3–4 amino acids is indicated with arrows. (**b**) Schematic of Voa1 and Ac45 proteins expressed from centromere plasmids in *voa1::H vma21QQ* yeast^24^. Ac45 proteins are either full length (intact-Ac45) or processed (cleaved-Ac45), with (shown) or without KKNN appended to the natural C terminus. Numbers indicate amino-acid residues. Residues mutated in Ac45 are shown. (**c**) Cleaved-Ac45 can substitute for Voa1 when a C-terminal dilysine motif is present. The *voa1::H vma21QQ* strain was transformed with plasmids coding for the indicated proteins (HA-tagged, diagrammed in (**b**)), Voa1QQ denotes Voa1 with K262Q and K263Q mutations. (**d**) The Y313C or E346K mutation in cleaved-Ac45-KKNN reduces V-ATPase function while protein levels are unaffected. Serial dilution growth test of *voa1::H vma21QQ* yeast expressing the indicated proteins tagged with HA. Restrictive growth is on rich medium adjusted to pH 7.5 and supplemented with 60 mM CaCl_2_. Membrane proteins prepared from the same cultures used in the growth test were analysed by western blot using anti-HA antibody to detect Voa1, cleaved-Ac45-KKNN and its mutant forms (band locations marked on the right, molecular mass (kDa) is indicated on the left. (**e**) Voa1 and cleaved-Ac45 require a C-terminal dilysine motif for ER localization. Fluorescent microscopy of live yeast cells showing DAPI stained DNA, GFP, the merged image of both, and cells viewed by differential interference contrast (DIC) to locate the vacuole as apparent indentation. The indicated proteins are N-terminally tagged with HA-GFP and expressed in *voa1::H vma21QQ* yeast cells. Exposure times for GFP images of cleaved-Ac45 were 10 × longer than for Voa1 or Voa1QQ. Perinuclear GFP fluorescence indicates ER localization. Mutated and non-mutated cleaved-Ac45-KKNN show the same localization. See also Supplementary Fig. 7.

Orthologs of Ac45 were readily identified by BLAST among the metazoa, including nematodes like *Caenorhabditis elegans*, but not outside of that taxon, leading to speculations about a role of Ac45 in specialized and complex vacuolar systems in multicellular organisms^25^. Nevertheless, the degree of sequence identity between vertebrate and invertebrate members of the protein family is relatively low, suggesting a high rate of sequence evolution as an alternative explanation for the inability to detect non-metazoan homologues. Using orthology prediction at the level of sequence profiles^32^, we detected two *S. cerevisiae* Ac45 homologues: Voa1 and Big1. Voa1's C-terminal transmembrane helix is significantly similar to the C terminus of Ac45 (*E*=9.1e−5) (Fig. 5a), while the sequence similarity of Ac45 to Big1 (*E*=3.6e−10) is mainly restricted to the N-terminal ∼250 amino-acid residues of Ac45 and therewith coincides with the part of Ac45 that is proteolytically cleaved by furin^9^,^30^. No significant sequence similarity could be detected in the dotted lines, or for the comparison of the C-terminal helix of Big1 with Ac45. Both proteins are located in the ER membrane, where Voa1 has been implicated in assembly of the V_0_ domain of the V-ATPase^24^ while Big1 is essential for beta 1,6 glucan synthesis^33^. Phylogenetic analysis shows that *BIG1* and *VOA1* appear the result of a gene duplication in the Saccharomycotina phylum of the Fungi (Supplementary Fig. 7B), indicating that both are orthologous to Ac45. Interestingly, the evolutionarily conserved residues between Voa1 and Ac45 are at a distance of 3–4 amino acids from each other (Fig. 5a) and concentrate on one side of the predicted transmembrane helix (Supplementary Fig. 7A), potentially forming a conserved interaction interface. Using iterative profile-based homology searches^34^, we confirmed the homology between the Ac45 and Voa1, Big1, and also detected homologues of Ac45 in major taxa of the eukaryotes, amoebozoa (*Dictyostelium discoideum*), brown algae (*Ectocarpus siliculosis*) and plants (Supplementary Fig. 7C). Ac45 orthologs in the model species *Arabidopsis thaliana* (AT3G13410) and *Schizosacharomyces pombe* (*S. pombe*) (SPCC306.06c) have, like Ac45, Voa1 and Big1, been observed in the ER^35^,^36^, indicating that the evolutionary origin of Ac45 lies at the root of the eukaryotes.

### Processed Ac45 functions in place of Voa1 in *S. cerevisiae*

In yeast, Vma21 and Voa1 are assembly factors of the V-ATPase V_0_ domain in the ER membrane. Both are retained in the ER via a C-terminal dilysine motif. When this motif is mutated to diglutamine, the resulting Vma21QQ or Voa1QQ protein is mislocalized to the vacuole with concomitant reduction in V-ATPase assembly and activity. The effect on V-ATPase assembly is cumulative, becoming most apparent when Voa1 is absent (*voa1::H*) or Voa1QQ is expressed in *vma21QQ* cells^24^.

Yeast lacking functional V-ATPase have a characteristic growth phenotype: they are unable to grow on medium buffered to pH 7.5, or medium containing elevated levels of calcium, or a combination of the two stressors^24^,^37^,^38^. Reduced V-ATPase function can be detected by reduced growth under any of these conditions. A growth assay on rich medium supplemented with 100 mM CaCl_2_ was used to assess the ability of human Ac45 to substitute for Voa1 (Fig. 5b,c). Full-length or processed Ac45 proteins with or without a dilysine motif (KKNN) appended to the C terminus were expressed in a *voa1::H vma21QQ* strain. Cells expressing full-length Ac45 grew poorly, comparable to cells transformed with empty vector or cells expressing Voa1QQ. Adding a dilysine motif to full-length Ac45 did not significantly improve growth. Conceivably, Ac45 function is dependent on proper processing of the protein, which might not be accomplished in yeast. Therefore, simulating a processed Ac45 protein^12^,^14^,^15^, the C-terminal half of Ac45 was expressed. This processed Ac45 was able to function in place of Voa1, but only when expressed with a dilysine motif (cleaved-Ac45-KKNN in Fig. 5c). By complementation, cleaved-Ac45-KKNN function is comparable to that of Voa1.

Growth assays were used next to measure the effect of three pathogenic Ac45 substitutions, Y313C, E436K or M428I (Fig. 5d, left panels). The mutations were introduced into cleaved-Ac45-KKNN and expressed in *voa1::H vma21QQ* yeast. Growth was tested on rich medium supplemented with 60 mM CaCl_2_ and buffered to pH 7.5. Cells expressing Y313C or E346K mutant protein showed a growth defect, with the E346K mutant most severely compromised, exhibiting reduced growth nearing that of yeast having no Voa1. The effect of the M428I substitution was less disruptive and appeared indistinguishable from non-mutated Ac45. To ascertain that the growth defect observed for the Y313C or E346K substitution was not the result of protein instability, membrane proteins from the cells used in the growth assay were examined by western blot (Fig. 5d, right panel). While Ac45 protein levels were lower than Voa1, levels for mutated Ac45 proteins were unchanged compared with non-mutated Ac45 protein. Therefore, reduced V-ATPase function observed for the Y313C or E346K mutation cannot be ascribed to decreased protein abundance.

Since both Voa1 and processed Ac45 require a dilysine motif for function, it is expected that, like Voa1, processed Ac45-KKNN is retained in the ER membrane, while absence of the motif would result in mislocalization to the vacuole. This was tested using GFP-tagged proteins (Fig. 5e, Supplementary Fig. 8). Though GFP-tagging slightly reduced fitness of processed Ac45-KKNN on restrictive medium (data not shown), dilysine-dependent ER localization was verified. Together with growth assay results, these results indicate that the human and yeast proteins function about equally well in V_0_ V-ATPase assembly in the ER.

## Discussion

Much has been learned about V-ATPase function and assembly by studies in yeast, however, studies in human have been very limited. The clinical symptoms resulting from Ac45 deficiency, mostly affecting the liver, immune system and brain, significantly differ from other known human genetic defects in various V-ATPase subunits. Disease mutations in subunits of the V_0_ domain result in, respectively, renal tubular acidosis with deafness (*ATP6V0A4* (MIM 602722) (refs 3, 4, 5)), osteopetrosis (*TCIRG1* or *ATP6V0A3* (MIM 259700) (refs 3, 6, 7)) and cutis laxa with abnormal glycosylation (*ATP6V0A2* (MIM 219200&278250) (ref. 8)). One subunit of the V_1_ domain is linked to human genetic disease (*ATP6V1B1* (MIM 267300)) resulting in renal tubular acidosis with deafness, while previously one of the V-ATPase assembly factors, VMA21, has been linked to XMEA (X-linked myopathy with excessive autophagy (MIM 310440) (ref. 29)). Very recently, mutations in *CCDC115* and *TMEM199*, as predicted orthologs of yeast V-ATPase assembly factors Vma22p and Vma12p, respectively, were identified in patients with liver disease, elevated alkaline phosphatase and cholesterol, mild abnormalities in copper metabolism and various degrees of cognitive impairment^39^,^40^. No immune dysfunction was noticed.

The combination of clinical symptoms as observed in Ac45-deficient patients is currently poorly understood since research on the functional roles of Ac45 has mainly been focused on neuroendocrine cells and osteoclasts. Certain specific symptoms could be related to other V-ATPase defects or known roles of the V-ATPase complex. These include deafness as reported in family 1, and also reported for ATP6V0A4 and ATP6V1B1 defects. In addition, electron microscopy of a liver biopsy of patient 3.1 suggested evidence for enhanced mitochondrial autophagy and muscle weakness with mildly elevated creatine kinase was found in some patients. This could suggest a partially overlapping disease mechanism with VMA21-deficient XMEA patients. Finally, in two of the patients, decreased enamelization of the teeth was reported, which could correspond with a recently reported role of V-ATPase-mediated acidification in enamelization^41^. Thus, likely at least some of the symptoms are related to V-ATPase dysfunction, which is supported by our studies on V-ATPase restricting growth conditions in yeast.

Immune and liver dysfunction have not yet been reported in genetic defects of the V-ATPase, although liver disease was recently described for defects in V-ATPase assembly factors TMEM199 and CCDC115 (refs 39, 40). The question is why these systems are affected. Possible explanations could include the tissue-specific processing of Ac45 or the existence of additional functions of Ac45 beyond pH regulation via its effect on the V-ATPase. Our studies in human hepatocytes show that Ac45, in contrast to what was observed in neuroendocrine cells, mostly localizes to the early secretory pathway. This is in agreement with the presence of mostly intact-Ac45 carrying non-processed high-mannose glycans. Further studies are needed to elucidate the mechanisms driving differential Ac45 glycosylation and processing in brain, liver and immune cells, since tissue-specific forms of Ac45 could suggest a possible mechanism for the tissue-restricted disease symptoms in Ac45-deficient patients.

Thus far, the relationship of Ac45 with immune deficiency has remained unnoticed. In view of the reported multiple functions of Ac45 in, for example, pH regulation and membrane trafficking and fusion^18^, many possible links exist. Acidification of phagolysosomes in, for example, macrophages is important for killing of internalized microorganisms, while antigen processing is also dependent on acidic pH. Our growth assay in yeast under conditions that are dependent on V-ATPase activity support the notion that the patients' phenotypes could be related to aberrant acidification due to dysfunction of the V-ATPase. Membrane trafficking and fusion events have not only been linked to V-ATPase function^42^,^43^,^44^ but also to Ac45 (refs 18, 43). These events are reported to be required for B-cell differentiation^45^, antigen processing^46^,^47^ and antibody production^45^. Thus, pathogenic mutations in *ATP6AP1* might affect B-cell function at all these levels, resulting in decreased levels of immunoglobulins and recurrent infections in our patients. The observed hypoglycosylation on serum transferrin in our patients might indicate hypoglycosylation on other proteins as well. Several membrane-bound proteins such as CD19 and CD40 that are involved in B-cell activation are glycosylated, and antigen recognition and antibody production by B cells require fucosylated IgG-BCR^48^. A glycosylation defect therefore may affect B-cell activation and thus antibody production. To find out which processes, that is, glycosylation, vesicular trafficking and fusion, or pH regulation are mainly affecting antibody production by a defective accessory subunit of the V-ATPase, further studies are required.

Previous studies have described an important role for Ac45 in intraorganellar pH regulation and membrane trafficking^10^,^14^,^15^,^16^,^30^. Identification of processed Ac45 as the functional ortholog of yeast V-ATPase assembly factor Voa1 only when the KKNN ER retention signal is present, indicates the importance of ER localization for its function in yeast, and provides a valuable model to further dissect the different functions and functional domains of Ac45. As the human ortholog lacks this dilysine motif, other mechanisms might account for retention of Ac45 to the ER of specific cell types such as liver cells. Our observation in liver cells that the Ac45 protein is mostly present in its unprocessed form, which in neuroendocrine cells appears to implicate ER localization^12^,^15^,^18^, combined with its observed steady-state localization in the early secretory pathway (ER, ERGIC) in hepatocytes, suggests that differential proteolytic processing might represent such a mechanism.

In summary, the identification of tissue-specific proteolytic processing of Ac45, and the availability of Voa1 mutant yeast as valuable model to further dissect the individual functions of Ac45 will facilitate future research to understand the functional roles and isoforms of Ac45 in the immune system, liver, muscle and brain and its relation to the V-ATPase in human. Screening for abnormal protein glycosylation in plasma of patients with hepatopathy and immune dysfunction with or without neurological symptoms provides a rapid way to identify additional individuals with ATP6AP1 deficiency.

## Methods

### Patients and glycosylation studies

Blood and fibroblasts of patients (clinical information in Table 1) were obtained for diagnostics of inborn errors of metabolism and used after informed consent from parents and/or treating physicians. Isoelectric focusing of serum transferrin for analysis of protein *N*-glycosylation defects and of serum apolipoprotein CIII for analysis of mucin type *O*-glycosylation defects were carried out as described before^49^. Plasma *N*-glycan profiling was performed by MALDI linear ion trap mass spectrometry as described^50^ using 10 μl of plasma. Briefly, serum was treated with PNGaseF, free *N*-glycans were permethylated, extracted and dried, purified and spotted onto a MALDI plate. Samples were dried and measured on a linear ion trap mass spectrometer. High resolution mass spectrometry of intact serum transferrin was performed as described^51^. Briefly, transferrin was immunopurified from 10 μl serum using anti-transferrin Sepharose beads. The elution with glycine-HCl pH 2.7 was neutralized by Tris-HCl pH 9.0 and was directly available for injection onto the nanoLC-C8-chip of the QTOF. Transferrin was eluted from the chip in a 10 min gradient of H2O and Acetonitrile, 0.1% formic acid. Charge distribution raw data were deconvoluted by Mass Hunter software to reconstructed mass spectra^51^.

### Next-generation sequencing

Genomic DNA was extracted from patient fibroblast according to the manufacturer's protocol using a Qiagen Mini kit (Qiagen, Hilden, Germany), and was checked for DNA integrity on agarose gels. Next-generation sequencing and analysis was performed as described^28^. In brief, exome enrichment was performed using the SureSelect Human All Exon 50 Mb Kit (Agilent, Santa Clara, CA), covering ∼21,000 genes. The exome library was sequenced on a SOLiD 5500xl sequencer (Life Technologies, Foster City, CA, USA). Colour space reads were iteratively mapped to the hg19 reference genome with the SOLiD LifeScope software version 2.1. Called variants and indels were annotated using an in-house annotation pipeline^52^,^53^ and common variants were filtered out based on a frequency of >0.5% in dbSNP (137) and a frequency of >0.3% in our in-house database of >1,300 exomes. Quality criteria were applied to filter out variants with <5 variant reads and <20% variation. Furthermore, synonymous variants, deep intronic, intergenic and UTR variants were excluded. Raw data of candidate variants were inspected using the Intergrative Genomic Viewer software (IGV browser) version 2.3.14 (2013) (ref. 54) (http://www.broadinstitute.org/igv/download). The putative consequences of the mutations found in the Ac45 protein were predicted using Sift (2009) (http://sift.jcvi.org/www/SIFT_aligned_seqs_submit.html), Mutation taster, (2014) (http://www.mutationtaster.org/) and Polyphen-2 (2012) (http://genetics.bwh.harvard.edu/pph2/) prediction programs.

### Sanger sequencing

Genomic DNA was extracted from fibroblast pellets or white blood cells from 10 patients and available family members. Primers (Supplementary Table 4) were designed to amplify the 10 exons of *ATP6AP1* (GenBank accession number NM_001183.4), including at least 50 bp of the flanking intronic regions. Standard PCR reactions were based on 1 μl DNA and 0.2 μl Platinum Taq polymerase (Invitrogen) in a total volume of 25 μl. Standard reaction conditions were 10 min at 95 °C, then 35 cycles of 30 s at 95 °C, 30 s at 60 °C and 1 min at 72 °C. The reaction was completed with a final elongation of 7 min at 72 °C. For the sequencing of the resulting PCR product, the BigDye Terminator Ready reaction cycle sequencing kit v.3.1 (Applied Biosystems) was used. Analysis of the results was performed on an ABI3100 Avant (Applied Biosystems).

### ATP6AP1 gene expression profiling in human tissues by qPCR

Total RNA from different human adult and fetal tissues was ordered from Stratagene Europe (Amsterdam, The Netherlands). All fetal tissues are from 20- or 21-week-old embryos after gestation. RNA was isolated using the NucleoSpin RNA II kit (Macherey-Nagel, Düren, Germany) according to the manufacturer's protocols. To remove residual traces of genomic DNA, the RNA was treated with DNase I (Invitrogen, Leek, The Netherlands) while bound to the RNA binding column. The integrity, concentration and purity of the RNA were assessed using agarose gel electrophoresis and spectrometry. Of all tissues, 5 μg of total RNA was transcribed into cDNA by using the iScript cDNA synthesis kit (Bio-Rad Laboratories, Hercules, CA, USA) according to the manufacturer's protocol. cDNA was purified by using the NucleoSpin extract II kit (Macherey-Nagel) according to the manufacturer's protocol.

Quantitative PCR quantifications were performed in on the equivalent of 12.5 ng total RNA input using the Sensifast SYBR no ROX qPCR kit (Bioline) and a Rotor-GeneTM 6000 real-time analyzer (Qiagen). qPCR program used was (2 min 95 °C (5 s 95 °C, 10 s 60 or 65 °C and 15 s 72 °C) × 40 cycles). Two pairs of intron-spanning *ATP6 AP1* primers were used. Primers used are listed in Supplementary Table 4. As reference transcripts, *GUSB* and *PPIB* were used.

qPCR data were analysed by using comparative quantitation and the relative Q-values of the genes of interest calculated by equalizing the lowest Ct value to 1. The normalization factor for the reference genes was determined using the GeNORM program (medgen.ugent.be/genorm) and used to normalize the Q-values. Individual experiments were performed in triplicate.

### Western blot analysis of Ac45 in cells and tissues

*B-cell isolation*. Buffy coats from two healthy donors, who gave written informed consent for scientific use of the buffy coats, were purchased from Sanquin Blood Bank, Nijmegen, The Netherlands. Peripheral blood mononuclear cells were isolated by density gradient centrifugation (Lymphoprep; Nycomed Pharma, Roskilde, Denmark). CD19+ B cells were positively selected using anti-CD19 magnetic microbeads (Miltenyi Biotec, Utrecht, The Netherlands). The isolated human donor B cells were lysed in cold lysis buffer (50 mM Hepes pH 7.4, 140 mM NaCl, 0.1% Triton-X100, 1% Tween-20, 0.1% deoxycholate supplemented with complete protease inhibitor mix (Roche Diagnostics)) to a final concentration of around nine million B cells per 20 μl lysisbuffer.

*Tissue lysates*. Tissue samples were powdered using a vessel and liquid nitrogen and subsequently lysed with cold lysis buffer to a final concentration of 100 mg tissue sample per 500 μl lysis buffer. All samples were incubated on ice for 15 min and repeatedly shortly vortexed during the incubation. Then, the homogenates were centrifuged at 14,000 RCF for 10 min, after which the supernatants were collected. Subsequent PNGase F treatment was done as follows: supernatant containing 21 μg of protein was incubated for 4–6 h at 37 °C with 2.5 μl of 500,000 U ml^−1^ of PNGase F (New England Biolabs) in a final volume of 34 μl containing 50 mM sodium phosphate buffer pH 7.5 (G7 buffer from PNGase F kit, New England Biolabs) and complete protease inhibitor mix (Roche Diagnostics). A second incubation was performed overnight at 37 °C after adding an additional 1 μl of PNGase F, 0.22 μl of G7 buffer, and 1 μl of protease inhibitor. The next day, the tubes were shortly centrifuged, SDS sample buffer was added, and the samples were boiled for 5 min at 99 °C. Samples were separated on 10% SDS–PAGE (7 μg of protein per lane) and the proteins were transferred to a 0.2-μm polyvinylidene difluoride membrane. After blocking in 5% milk in PBS+1% Tween-20, the membrane was incubated overnight at 4 °C with primary antibody rabbit anti-mouse Ac45 polyclonal #49 antiserum (directed towards A271-T283 and L443-I457 of mouse Ac45, kindly provided by Dr J. Creemers, Catholic University, Leuven, Belgium) at a dilution of 1:5,000 in blocking buffer. Goat-anti rabbit-HRP secondary antibody (Dako, P0448) at a dilution of 1:5,000 in 2.5% milk in PBS-1% Tween-20 were used for ECL detection. To check for protein loading, the membrane was incubated with mouse anti-GapdH monoclonal antibody (Ab8245-100, Abcam) at a dilution of 1:2,000 in 3% BSA in PBS/0.1% Tween-20 for 1 h at room temperature. Incubation with the secondary antibody (Goat-anti-mouse-HRP, Dako P0447) was for 1 h at room temperature at a dilution of 1:5,000 in 2.5% milk in PBS-1% Tween-20. Chemoluminescent signals was detected using ECL (Pierce).

### Biochemical studies in immortalized human hepatocytes

IHH^55^ were cultured in gelatin-coated culture flasks in Williams medium E supplemented with 10% FCS, 0.022 U ml^−1^ insulin and 0.045 μg ml^−1^ dexametasone. Culturing was done at 37 °C under an atmosphere of 5% CO_2_. IHH cell cultures were tested negative for mycoplasm. For Ac45 expression, IHH cells were transfected with a hsAc45/pcDNA3 construct using Lipofectamine LTX (Invitrogen) according to manufactures' guidelines.

For immunofluorescence assays, cells were cultured on gelatin-coated cover slips for 3 days and fixed for 1 h by 4% paraformaldehyde in PBS at room temperature. After blocking of residual parafomaldehyde with 50 mM NH_4_Cl in PBS, cells were permeabilized using 0.1% Triton-X100 in PBS (PBS-T) and incubated with anti-Ac45 antibody (1:1,000) and mouse-anti-EEA1 (BD Biosciences, 1:200), mouse-anti-M6PR (Abcam, 1:200), mouse-anti-Sec31a (Santa Cruz, 1:200), mouse-anti-GM130 (BD Transduction Laboratories, 1:400), mouse-anti-PDI (Stressgen, 1:500), mouse-anti-Rab11 (BD Biosciences, 1:100), mouse-anti-ERGIC53 (Santa Cruz, 1:100) and goat-anti-TGN38 (Santa Cruz, 1:100) antibodies in 1% BSA in PBS-T (blocking buffer) for 12 h at 4 °C. After washing with PBS-T, cells were incubated for 45 min at room temperature with secondary antibodies Goat-anti-rabbit-Alexa488 and Goat-anti-mouse-Alexa568 or Donkey-anti-rabbit-Alexa488 combined with Donkey-anti-goat-Alexa568 at a dilution of 1:200 in blocking buffer. Hereafter, cells were washed with PBS-T, PBS, MilliQ water and dehydrated using methanol and subsequently mounted in Mowiol containing 2.5 μM DAPI. Imaging was performed using an Olympus FV1000 confocal laser scanning microscope using a × 63 oil objective. All images were captured with an aspect ratio of 1,200 × 1,200 using the FluoView version 4.1 software at a scanning speed of 12.5 μs per pixel. Image analysis was performed using Fiji software. Relative fluorescence intensities were calculated over a 10-μm cross section by setting the highest measured value to 100.

### Newly synthesized protein labelling experiments

Two days prior to labelling, IHH cells were seeded into gelatin-coated 12-well plates at a density of 2 × 10^5^ cells per well. Cells were washed using PBS and starved in starvation medium (DMEM without methione and cysteine, supplemented with 10% dialysed FCS, 0.022 U ml^−1^ insulin and 0.045 μg ml^−1^ dexametasone) for 1 h. Then, cells were incubated for a 30-min pulse period in starvation medium containing 0.1 mCi ml^−1^ EasyTag EXPRESS ^35^S protein labelling Mix (PerkinElmer). For analysis of the *N*-glycosylation of Ac45, the 30-min pulse was carried out in the presence of tunicamycin. Following the pulse period, cells were washed with PBS and lysed in lysis buffer. For immunoprecipitation, lysates were incubated with anti-Ac45 antibodies (1:500) in lysis buffer supplemented with 0.8% SDS for 12 h at 4 °C. Immune complexes were precipitated by Protein A-sepharose (GE Healthcare Life Sciences) and resolved on 10% SDS–PAGE. EndoH (New England Biolabs) and PNGaseF (New England Biolabs) treatment was performed on immunoprecipitated Ac45 protein according to manufacturer's guidelines. Radiolabelled proteins were visualized by fluorography.

### Transmission electron microscopy

The tissue cylinder was immediately immersed in cold 2.5% glutaraldehyde in 0.1 M Cacodylate buffer (pH 7.4) for 2 h., post fixed in 1% osmium tetroxide for 1 h., dehydrated through ethanol series and embedded in epoxy resin. Semi-thin sections (1 μm) stained with 1% toluidine blue were used for orientation, cell identification and gross histopathological changes (that is, lipid droplets). Ultrathin sections (60 nm) were cut with diamond knives and either left unstained or stained with uranyl acetate and lead citrate. Sections from three blocks were placed on 300-mesh copper grids and viewed and photographed with JEOL JEM 100SX and 100CX electron microscopes, operated at 80 kV.

### Bio-informatics studies

For sequence-profile-based homology searching, we used HHpred^56^ with three PSI-Blast iterations to create the initial sequence profiles. The initial profiles were based either on Ac45 after which that profile was compared with the profiles of *S. cerevisiae* and *S. pombe*, on Voa1 after which that profile was compared to the profiles of *H. sapiens*, or with Big1 after which that profile was compared with the profiles of *H. sapiens*. The expectation values (*E*-values) obtained via homology detection using HHpred^55^ with the sequence profile based on Ac45 (arrows from Ac45 outwards) and for the reciprocal searches with Voa1/Big1 (arrows towards Ac45) are indicated in Fig. 5a.

### Yeast strain and plasmids

Standard molecular biology protocols for *E. coli* and yeast manipulations were followed^57^. The *voa1::H vma21QQ* yeast strain used in this study is isogenic with SF838-1Dα (*MATα ura3-52 leu2-3,112 his4-519 ade6 pep4-3 gal2*) (ref. 58) and additionally has *voa1::Hyg*^*r*^ *vma21QQ::HA* (MRY5) (ref. 24). Plasmids used are listed in Supplementary Table 5. All pMR plasmids have in common the pRS316 vector^59^, 821 bp of *VOA1* 5′ UTR sequence, coding sequence for Voa1 signal peptide (M1–A24), and the first Voa1 amino acid after signal cleavage (D25) followed by a single HA tag or HA-GFP. Sequence coding for C-terminal KKNN of Voa1 was added to Ac45 where indicated. All pMR plasmids have 245 bp of *VOA1* 3′ UTR flanking sequence.

A pMR072 (ref. 24) derived precursor plasmid, pMR124, was prepared where Voa1 amino acids I218–I261 are replaced with human Ac45 F421–V470 by using yeast codon optimized oligonucleotides and PCR. pMR1210 was prepared from pMR124 by replacing Voa1 S26–S217 with human Ac45 Q43–S420, PCR amplified from pOTB7/ATP6AP1 (MGC cDNA clone, IMAGE:3506925, Thermo Scientific), using the FastCloning technique^60^. Thus pMR1210 encodes full-length Ac45-KKNN. KKNN was deleted from the C-terminal end of Ac45 in pMR1210, giving pMR1211. The N-terminal half of Ac45 (Q43-V250) was deleted in pMR1210 and pMR1211, resulting in pMR1213 and pMR1214, respectively. Primers designed to produce mutation Y313C, E346K or M428I and pMR1213 template were used to make pMR1213Y313C, pMR1213E346K or pMR1213M428I. pMR092 was made by inserting mGFP5(S65T)^61^ into pMR072 between the HA tag and Voa1 S26 using PCR techniques. Inverse PCR with primers encoding K262Q and K263Q mutations and pMR092 template produced pMR1312. pMR1303 and pMR1304 were made from pMR1213 and pMR1214, respectively, by inserting GFP immediately after the HA tag. E346K and M428I mutations were made in pMR1303, resulting in pMR1303E346K and pMR1303M428I, respectively. Coding sequence region in all plasmid constructs was verified by DNA sequencing.

### Yeast growth assay

Liquid cultures of yeast strains were grown overnight at 30 °C in synthetic minimal medium plus dextrose (SD) supplemented to select for plasmid (-Ura), diluted to 0.4 OD_600_ per ml in rich medium (YEPD) buffered to pH 5 with 50 mM succinate/phosphate, and grown until densities reached 1 OD per ml. Cells were pelleted and suspended to 0.8 OD per ml in H_2_O. This suspension and serial 1:8 dilutions were spotted onto YEPD pH 5 and restrictive medium (100 mM CaCl_2_ in pH-unadjusted YEPD, or 60 mM CaCl_2_ in YEPD buffered to pH 7.5 with 50 mM HEPES). Growth was recorded after 46 h incubation at 30 °C.

### Western blot analysis of yeast proteins

Yeast membrane proteins were prepared from glass-bead lysates by centrifugation at 13,000*g* as described^24^. The pelleted membrane fraction was solubilized in sample buffer (8 M urea, 5% SDS, 40 mM Tris-HCl pH 6.8, 5% β-mercaptoethanol, 0.01% bromophenol blue) to 100 OD_600_ equivalents per ml, and 0.5 OD equivalents separated by SDS–PAGE. Proteins were transferred to nitrocellulose membrane and analysed by western blot using a 1:300 dilution of monoclonal anti-HA primary antibody (BioLegend) and a 1:15,000 dilution of goat anti-mouse secondary antibody labelled with IRDye 800CW (LI-COR). Blocking of non-specific binding and incubation with antibody was done in 5% non-fat dry milk, 0.1% TWEEN 20, 0.5 M NaCl, 20 mM Tris-HCl pH 7.5. Wash steps after antibody incubation were in the same buffer but without milk. Before imaging (Odyssey F_c_ Imager, LI-COR), a final wash was done in 10 mM Tris-HCl pH 7.5, 150 mM NaCl.

### Live cell imaging of yeast cells

Yeast cells expressing GFP-tagged proteins were grown overnight in SD-Ura, diluted to 0.2 OD_600_ per ml in SD-Ura containing 2.5 μg ml^−1^ DAPI (Sigma) and grown until 0.4 OD_600_ per ml. Cells were collected by centrifugation, and 1.5 μl of cell pellet was added to an equal volume of molten 1% agarose on a glass slide for live cell imaging. Images were acquired with an Axioplan 2 fluorescence microscope (Carl Zeiss) using a × 100 objective. Axiovision (Carl Zeiss) and ImageJ (http://imagej.nih.gov/ij) software were used for image evaluation, and Photoshop CS4 for image manipulation.

### Data availability

The authors declare that all data supporting the findings of this study are available within the article and its Supplementary Information files.

## Additional information

**How to cite this article:** Jansen, E. J. R. *et al*. *ATP6AP1* deficiency causes immunodeficiency with hepatopathy, cognitive impairment and abnormal protein glycosylation. *Nat. Commun.* 7:11600 doi: 10.1038/ncomms11600 (2016).

## Supplementary Material

Supplementary Information Supplementary Figures 1-8, Supplementary Tables 1-5 and Supplementary References

## Figures and Tables

**Table 1:**
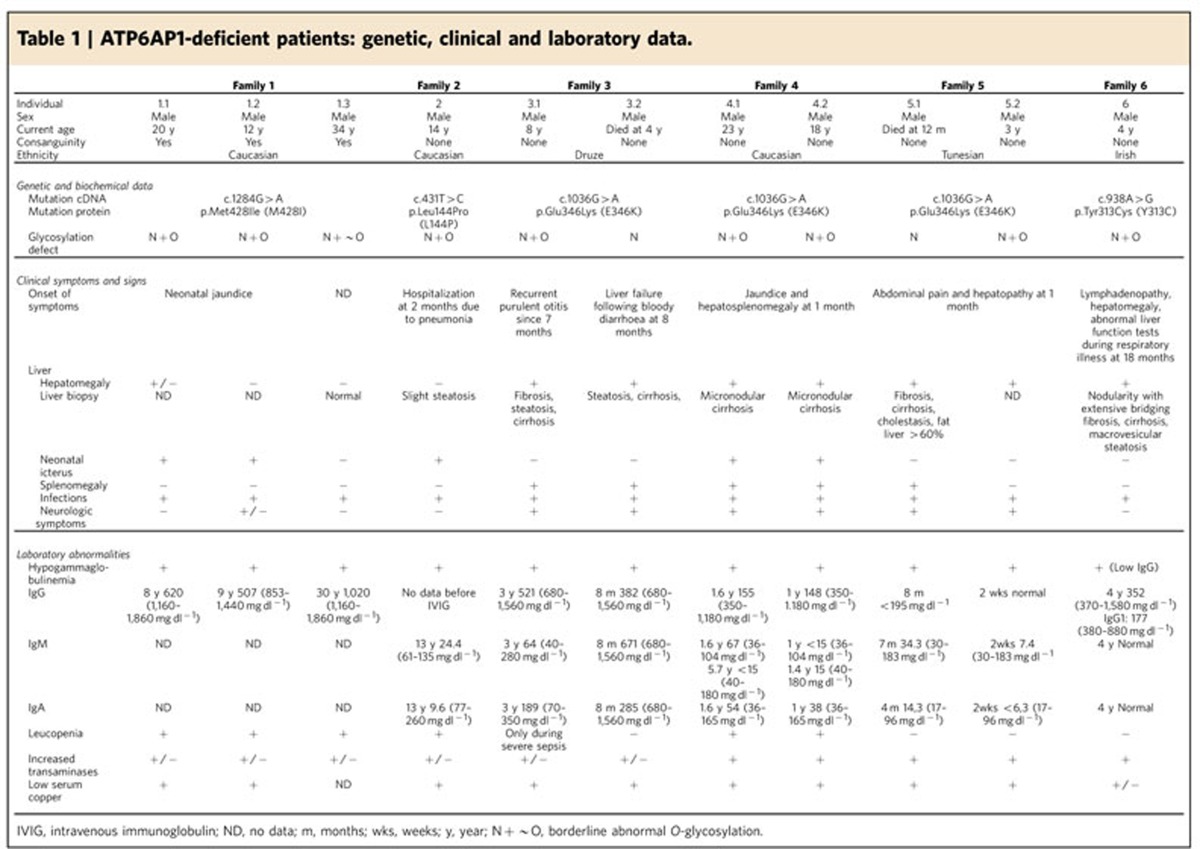
ATP6AP1-deficient patients: genetic, clinical and laboratory data.
