# Supplementary material for: ATP6AP1 deficiency causes an immunodeficiency with hepatopathy, cognitive impairment and abnormal protein glycosylation
Source: Nat Commun. 2016 May 27;7:11600. doi: 10.1038/ncomms11600 (PMC4894975; doi:10.1038/ncomms11600)
Supplement: Supplementary Information — Supplementary Figures 1-8, Supplementary Tables 1-5 and Supplementary References [file ncomms11600-s1.pdf]

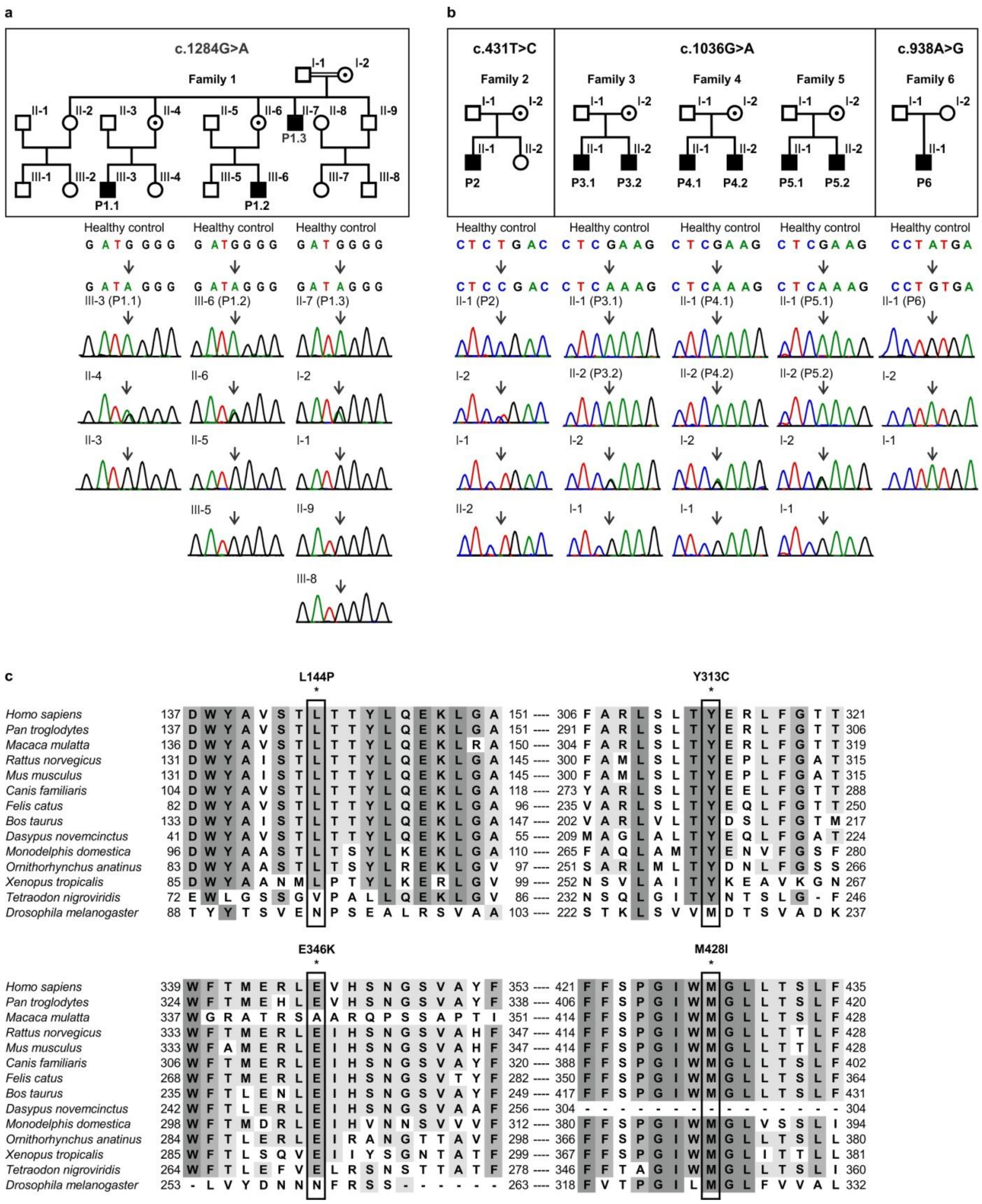

Supplementary Fig. 1. ***ATP6AP1*** **gene mutations in all families.**

(a) Sequence profiles show the 1284G>A change in patients P1.1, P1.2 and P1.3. Subjects II-4, II-6 and I-2 are the mothers of the patients who carry the mutation in heterozygous state. Fathers (subjects I-1, II-3, II-5, and II-9) do not carry the mutation and are hemizygous wild type. Individuals III-8 and III-5 were also sequenced and were hemizygous wildtype. DNA of individuals II-1, II-2, III-1, and III-2 was not available for sequencing. **(b)** *ATP6AP1* gene mutations in families 2-6. Mutation c.431T>C in family 2 is only found in hemizygous state in patient P2, and in heterozygous state in the mother, indicated as I-2. The c.1036A>G mutation is found in heterozygous state in all mothers of families 3-5. Mutation c.938A>G is only found in patient 6, and not in the parents. The *de novo* nature was confirmed by STR marker analysis (data not shown). In all families, fathers are hemizygous wild type. **(c)**

Conservation is shown of the substituted amino acids of Ac45 as found in families 1-6. Substituted amino acids are indicated with an asterisk (*).

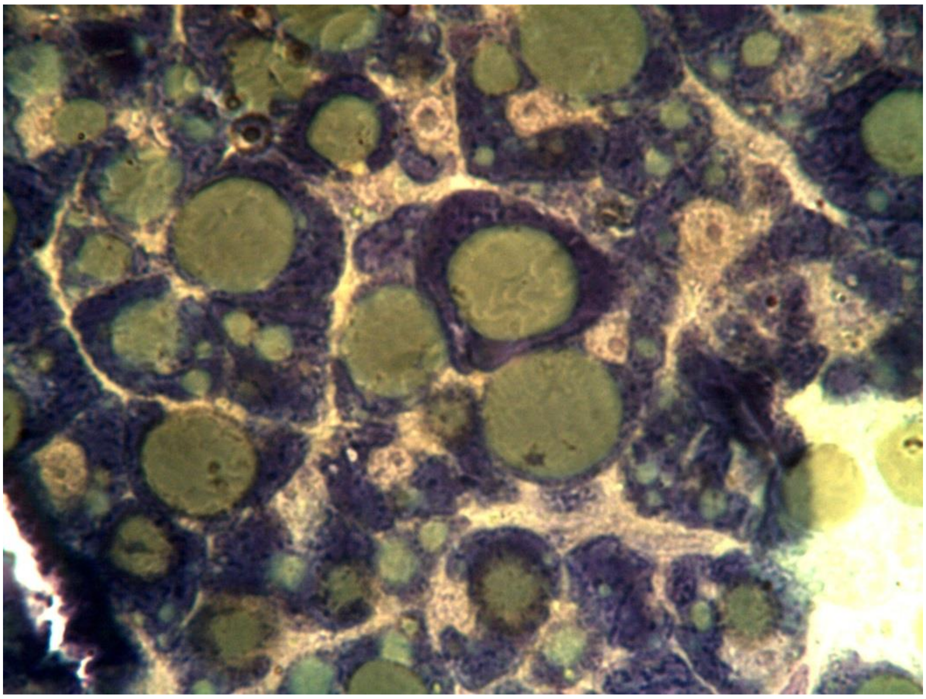

Supplementary Fig. 2. Liver biopsy of patient 3.1
Light microscopy reveals severe steatosis of hepatocytes. Shown is a toluidine blue stain, x 1,000.

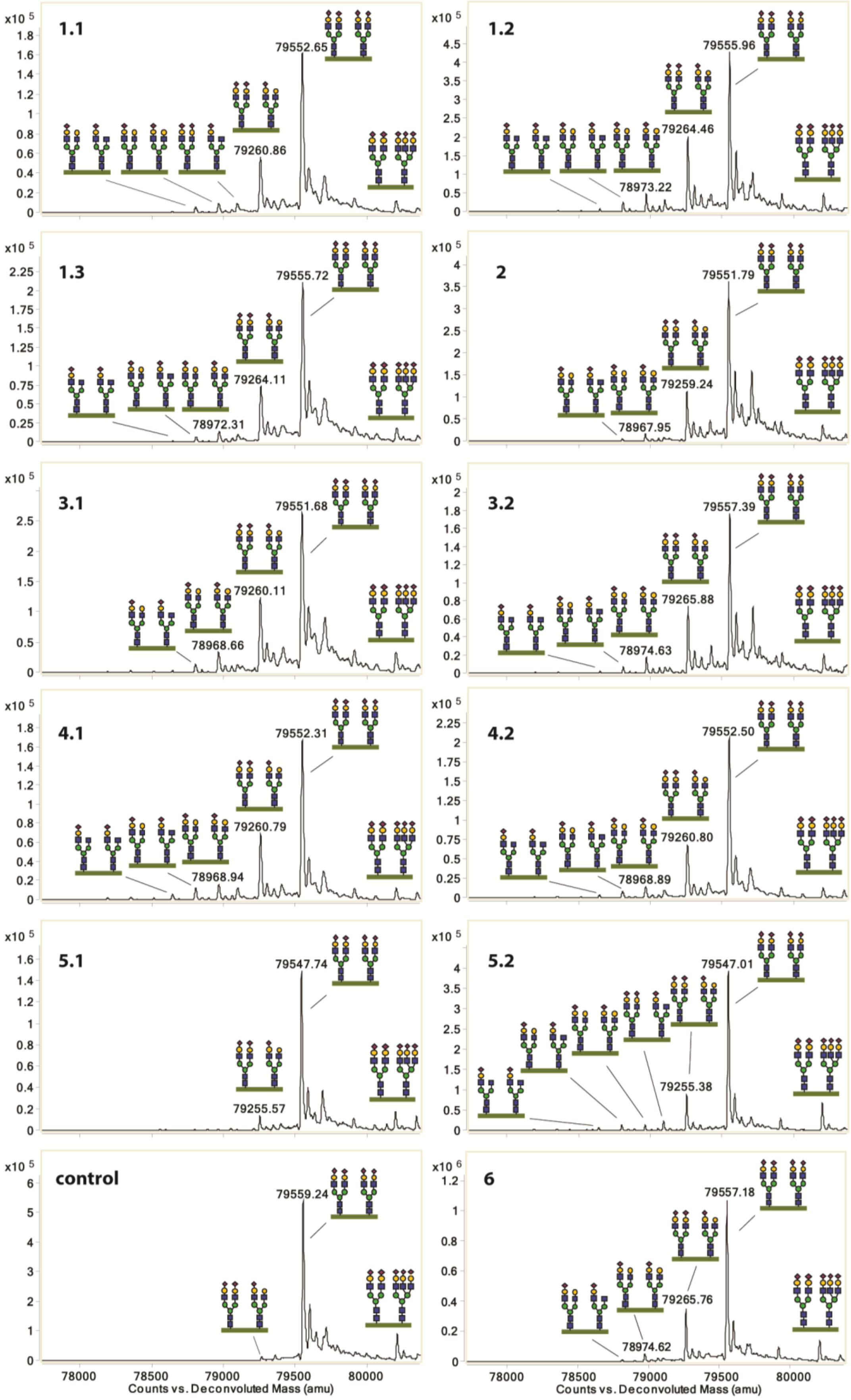

Supplementary Fig. 3. High resolution mass spectra of intact serum transferrin of all patients

Similar peaks of truncated structures lacking galactose and sialic acids were observed for all patients, although in variable intensity.

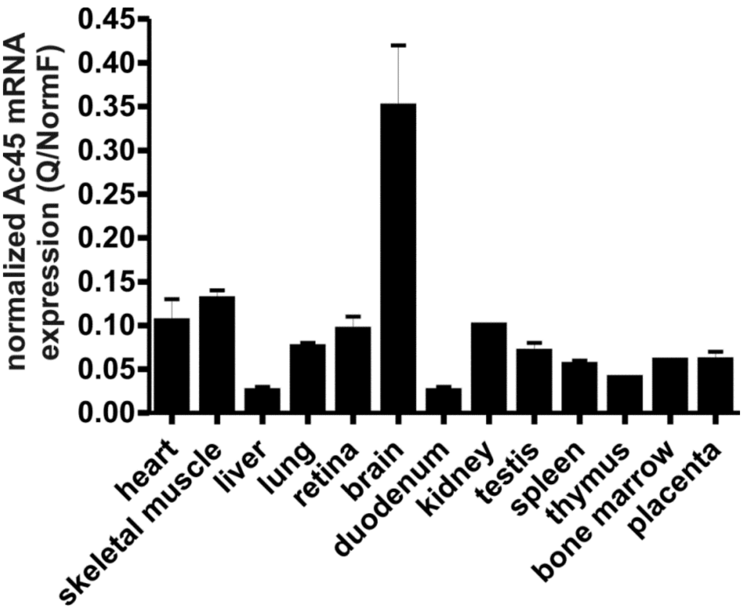

Supplementary Fig. 4. Analysis of Ac45 mRNA expression levels in different human adult tissues

qPCR quantifications were performed on the equivalent of 12.5 ng total RNA. Primers used are listed in Supplementary **Table 4**. As reference transcripts, *GUSB* and *PPIB* were used. qPCR data were analyzed by using comparative quantitation and the relative Q-values of the genes of interest calculated by equalizing the lowest Ct value to 1. The normalization factor (NormF) for the reference genes was determined using the GeNORM program (medgen.ugent.be/genorm) and used to normalize the Q-values. Error bars represent S.E.M.

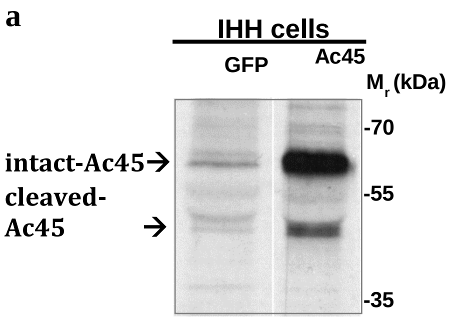

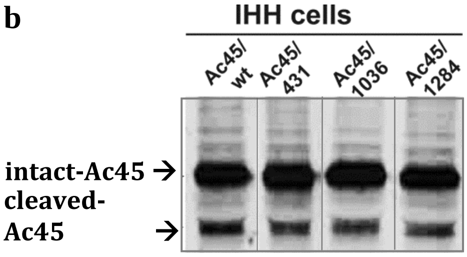

Supplementary Fig. 5. Analysis of newly-synthesized human (mutant) Ac45 in human hepatocytes (IHH)

(a) Cells were transfected with either GFP or Ac45 construct, pulsed for 30-min with ^{35}S-methionine/cysteine, and Ac45 was immunoprecipitated using the anti-Ac45 antibody (see Experimental Procedures). Immunoprecipitates were resolved by 10% SDS-PAGE. **(b)** To study protein stability, mutant constructs of Ac45 (c.431T>C, c.1036G>A or c.1284G>A) were transfected in IHH cells and analysed after 30-min ^{35}S labeling and immunoprecipitation of Ac45.

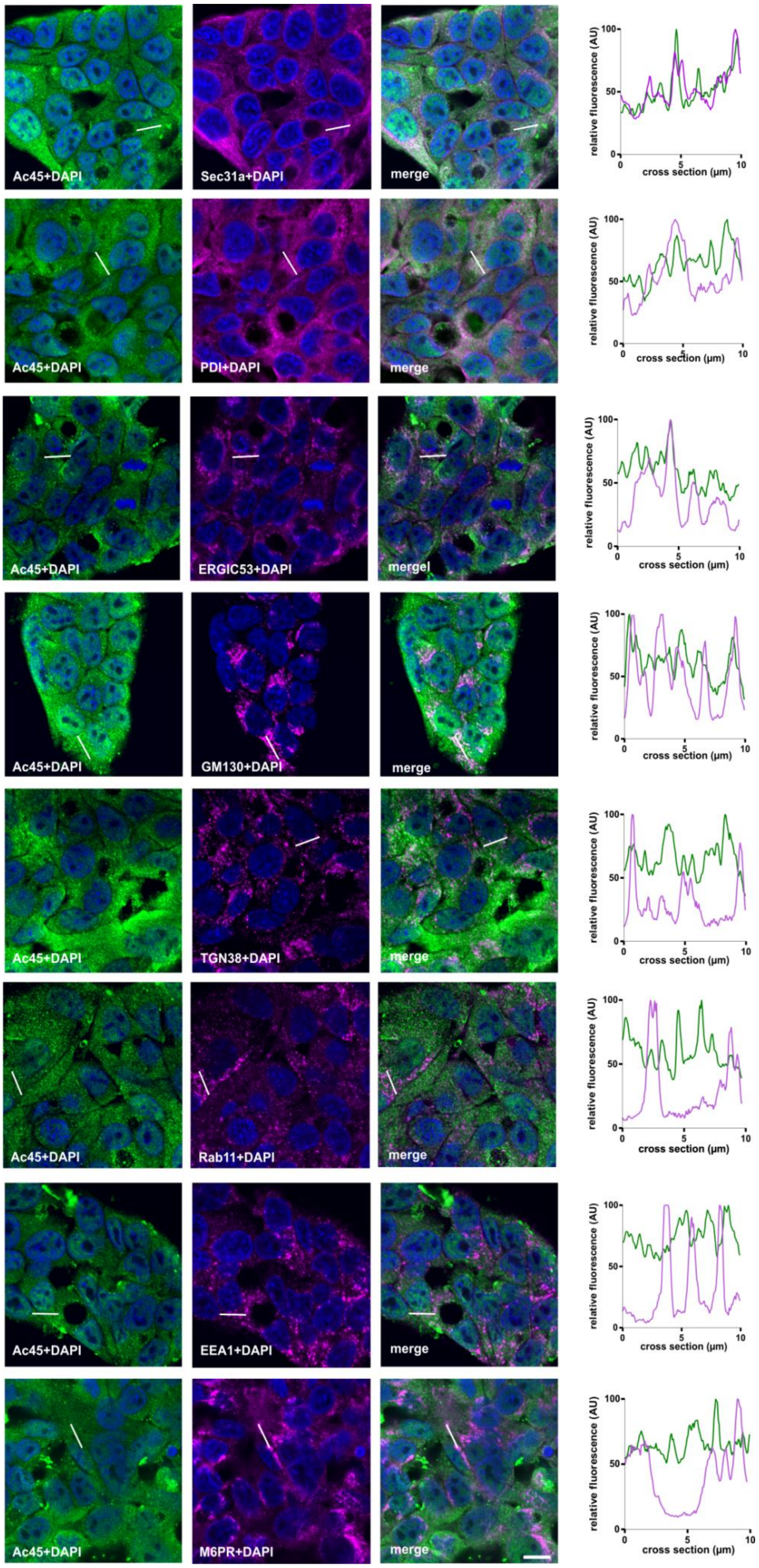
Ac45+DAPI
Sec31a+DAPI
merge
PDI+DAPI
ERGIC53+DAPI
mergel
GM130+DAPI
TGN38+DAPI
Rab11+DAPI
EEA1+DAPI
M6PR+DAPI
relative fluorescence (AU)
cross section (µm)

Supplementary Fig. 6. Ac45 in IHH cells is mainly localized to the early secretory pathway

IHH cells were stained with the anti-Ac45 antibody (green) together with antibodies against different organelle markers (magenta). Markers used included ER marker protein disulfide-isomerase (PDI), the COPII vesicle marker Sec31a, ER-Golgi intermediate compartment marker ERGIC-53, the Golgi matrix protein 130 (GM130), *trans*-Golgi network integral membrane protein 38 (TGN38), endocytic recycling compartment/TGN marker Rab11, early endosome antigen-1 (EEA1) and late endosomal marker Mannose-6-phosphate receptor (M6PR). A cross section of 10 µm was used to measure the relative fluorescence of Ac45 and the respective marker. Bar = 10 µm

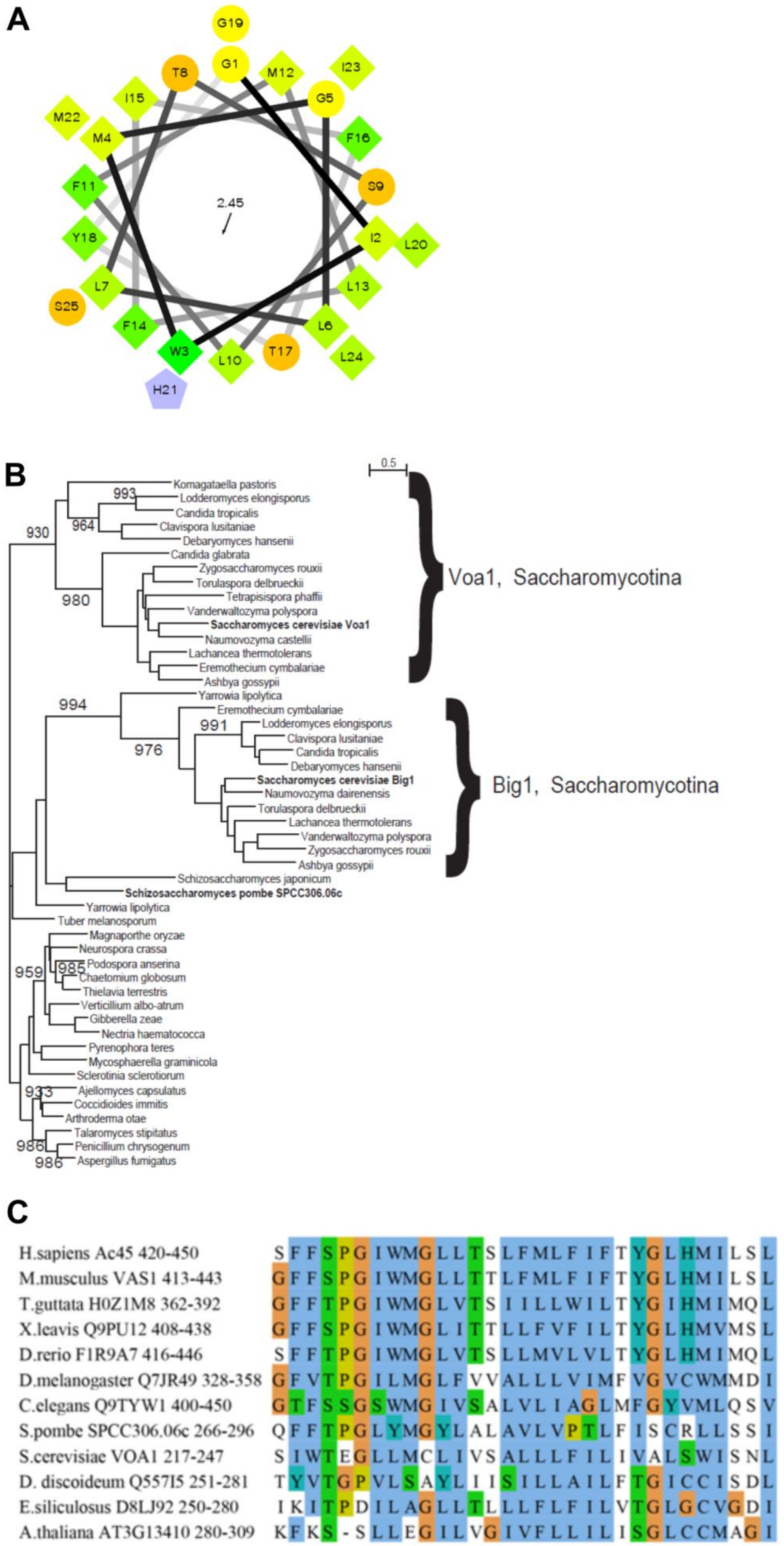
A
2.45
B
0.5
Voa1, Saccharomycotina
Big1, Saccharomycotina
Komagataella pastoris
Lodderomyces elongisporus
Candida tropicalis
Clavispora lusitaniae
Debaryomyces hansenii
Candida glabrata
Zygosaccharomyces rouxii
Torulaspora delbrueckii
Tetrapisispora phaffii
Vanderwaltozyma polyspora
Saccharomyces cerevisiae Voa1
Naumovozyma castellii
Lachancea thermotolerans
Eremothecium cymbalariae
Ashbya gossypii
Yarrowia lipolytica
Eremothecium cymbalariae
Lodderomyces elongisporus
Clavispora lusitaniae
Candida tropicalis
Debaryomyces hansenii
Saccharomyces cerevisiae Big1
Naumovozyma dairenensis
Torulaspora delbrueckii
Lachancea thermotolerans
Vanderwaltozyma polyspora
Zygosaccharomyces rouxii
Ashbya gossypii
Schizosaccharomyces japonicum
Schizosaccharomyces pombe SPCC306.06c
Yarrowia lipolytica
Tuber melanosporum
Magnaporthe oryzae
Neurospora crassa
Podospora anserina
Chaetomium globosum
Thielavia terrestris
Verticillium albo-atrum
Gibberella zeae
Nectria haematococca
Pyrenophora teres
Mycosphaerella graminicola
Sclerotinia sclerotiorum
Ajellomyces capsulatus
Coccidioides immitis
Arthroderma otae
Talaromyces stipitatus
Penicillium chrysogenum
Aspergillus fumigatus
C
H.sapiens Ac45 420-450
M.musculus VAS1 413-443
T.guttata H0Z1M8 362-392
X.leavis Q9PU12 408-438
D.rerio F1R9A7 416-446
D.melanogaster Q7JR49 328-358
C.elegans Q9TYW1 400-450
S.pombe SPCC306.06c 266-296
S.cerevisiae VOA1 217-247
D. discoideum Q557I5 251-281
E.siliculosus D8LJ92 250-280
A.thaliana AT3G13410 280-309

Supplementary Fig. 7. Homology prediction of Voa1 and Big1
(a) Helical wheel projection of the 10 most strongly conserved positions in the C-terminal transmembrane helix of homologs of Ac45. The projection starts at the first amino acid "before" the transmembrane region (G1) and continues until the first amino acid after the helix (S25). Most of the conserved residues are on one side of the helix, suggesting a conserved interaction interface. Conservation was calculated with JACKHMMER and the helical wheel projection was drawn with the tool at http://rzlab.ucr.edu/scripts/wheel/wheel.cgi. **(b)** Phylogeny of the ATP6AP1/Big1/Voa1 protein family among Fungi. Species that have both Voa1 and Big1 are all part of the Saccharomycotina clade. Species that only have a single copy of that gene fall outside that of the Saccharomycotina clade, suggesting that the duplication that gave rise to Big1 and Voa1 happened early in the evolution of the Saccharomycotina. The neighbor-joining phylogeny is based on a ClustalX alignment using the identity matrix for sequence similarity and correcting for multiple substitutions. Genbank identifiers for the sequences used are indicated with the species names. **(c)** Alignment of the C–terminal transmembrane helix, and its flanking aminoacids of ATP6AP1 homologs among representative species of the various eukaryotic taxa. The homologs were detected with JACKHMMER. With each sequence its uniprot identifier is indicated. Full species names are: Homo sapiens, Mus musculus, Taeniopygia guttata, Xenopus laevis, Danio rerio, Drosophila melanogaster, Caenorhabditis elegans, Schizosaccharomyces pombe, Saccharomyces cerevisiae, Dictyostelium discoideum, Ectocarpus siliculosus, Arabidopsis thaliana.

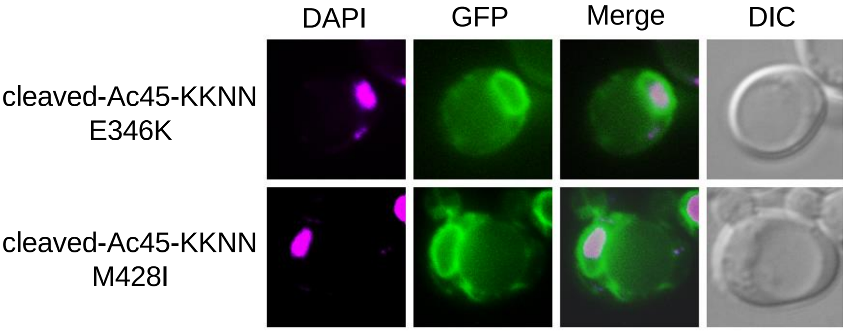

Supplementary Fig. 8. ER localization of mutant forms of cleaved-Ac45-KKNN
Fluorescent microscopy of live yeast cells showing DAPI stained DNA, GFP, the merged image of both, and cells viewed by differential interference contrast (DIC) to locate the vacuole as apparent indentation. The indicated proteins are N-terminally tagged with HA-GFP and expressed in *voa1::H vma21QQ* yeast cells. ER localization was also observed for the cleaved-Ac45-KKNN Y313C mutant (not shown). Perinuclear GFP fluorescence indicates ER localization.

Supplementary Table 1: Candidate variants found by exome sequencing in patient 1.1

Chr.	Genomic position [hg19]	Reference nucleotide	Variant nucleotide	Coverage	variant reads	% variation	SNP ID/ SNP state	In-house db	Gene component	Gene	Isoform ID	Strand	Protein change	cDNA change	phyloP	Grantham Score
Homozygous variants [sorted by phyloP]:																
**chrX**	153664108	G	A	65	65	100		0	Exon	ATP6AP1	NM_001183	+	p.Met428Ile	c.1284G>A	5.11	10
Compound heterozygous variants [sorted by phyloP]:																
**chr1**	152732227	C	T	20	5	25		0	Exon	KPRP	NM_001025231	+	p.Gln55*	c.163C>T	3.44	1000
**chr1**	152733472	C	T	195	94	48	rs141557248/ Same SNP	6	Exon	KPRP	NM_001025231	+	p.Pro470Ser	c.1408C>T	0.75	74

Chr. = Chromosome; SNP ID = dbSNP137 identifier; In-house db = In-house database count in >1300 exomes; PhyloP = 46 vertebrate PhyloP score

Supplementary Table 2: Quantification of Transferrin and ApoCIII isofocusing profiles

Patient ID	T0	T1	T2	T3	T4	T5	T6	Age	ApoCIII-0	ApoCIII-1	ApoCIII-2
1.1	1.2	2.7	11.1	27.4	42.3	11.5	3.8	9 yr	5.7	82.8	11.5
1.2	2.0	3.6	13.6	26.8	38.0	13.2	2.8	8 yr	5.6	77.8	16.6
1.3	2.0	3.2	11.1	22.7	40.1	17.4	3.5	29 yr	2.1	75.6	22.3
2	0.0	3.1	8.0	25.5	47.3	13.3	2.9	8 yr	6.6	77.7	15.7
3.1	2.5	6.4	17.9	23.5	32.0	14.3	3.4	3 yr	4.0	88.2	7.8
3.2	4.8	4.8	12.1	20.1	35.5	17.7	4.4	1 yr	5.8	61.4	32.8
4.1	1.3	5.3	14.7	27.1	40.8	9.5	1.4	21 yr	5.5	83.8	10.7
4.2	1.5	4.1	12.3	26.3	45.3	9.2	1.4	16 yr	6.9	72.7	20.4
5.1	1.7	2.5	8.1	16.2	47.0	20.7	3.8	1 yr	4.6	38.2	57.2
5.2	1.7	4.0	11.1	19.4	47.6	13.5	2.7	3 mo	5.6	83.9	10.5
6	1.0	1.7	9.4	26.0	46.1	13.6	2.3	2 yr	4.6	78.5	16.9
Control range	0-3.2	0-5	3.3-7.6	4.9-10.6	47.3-62.7	18.7-31.5	3.2-7.8	**0-1 yr**	0.2 - 4.5	42.7 - 69.8	26.2 - 56.7
								**1-18 yr**	1.4 - 9.5	48.5 - 75.2	21.0 - 45.8
								**>18 yr**	1.8 - 7.6	47.4 - 73.6	20.2 - 46.5

Supplementary Table 3: Mass and abundance of transferrin isoforms

	Peak number / Structure	1	2	3	4	5	6	7
		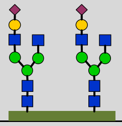	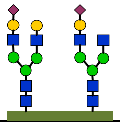	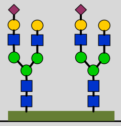	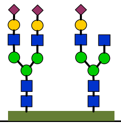	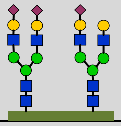	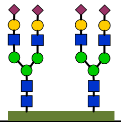	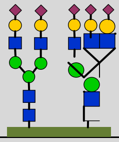
**Control**	Mass (amu)	n.d.	n.d.	n.d.	n.d.	79267.23	79559.24	80216
	Height (%)	n.d.	n.d.	n.d.	n.d.	2.2	100.0	16.5
**1.1**	Mass (amu)	78643.79	78807.33	78968.79	79100.03	79260.86	79552.65	80208.94
	Height (%)	0.7	3.2	5.7	5.7	34.7	100.0	7.3
**1.2**	Mass (amu)	78648.98	78811.2	78973.22	79104.42	79264.46	79555.96	80212.26
	Height (%)	1.7	6.1	10.9	7.2	47.2	100.0	11.5
**1.3**	Mass (amu)	78647.1	78810.31	78972.31	79104.89	79264.11	79555.72	80212.88
	Height (%)	0.5	3.0	6.5	4.6	34.8	100.0	8.7
**2**	Mass (amu)	n.d.	78804.91	78967.95	79097.46	79259.24	79551.79	80208.5
	Height (%)	n.d.	1.4	4.3	2.2	31.1	100.0	10.2
**3.1**	Mass (amu)	78643.74	78806.3	78968.66	79100.26	79260.11	79551.68	80208.45
	Height (%)	1.2	4.9	12.5	4.6	46.8	100.0	12.9
**3.2**	Mass (amu)	78649.94	78812.32	78974.63	79105.89	79265.88	79557.39	80213.93
	Height (%)	1.0	3.7	9.8	3.6	42.0	100.0	11.9
**4.1**	Mass (amu)	78645.54	78807	78968.94	79101.03	79260.79	79552.31	80209.09
	Height (%)	3.2	7.0	9.5	7.4	41.0	100.0	7.0
**4.2**	Mass (amu)	78645.22	78807.07	78968.89	79102.68	79260.8	79552.5	80209.42
	Height (%)	1.7	4.1	6.9	4.6	33.3	100.0	5.8
**5.1**	Mass (amu)	n.d.	n.d.	78965.18	n.d.	79255.57	79547.74	80203.7
	Height (%)	n.d.	n.d.	0.7	n.d.	9.2	100.0	11.7
**5.2**	Mass (amu)	78639.89	78801.66	78964.17	79093.44	79255.38	79547.01	80203.34
	Height (%)	1.8	3.5	3.5	6.2	22.7	100.0	17.3
**6**	Mass (amu)	78649.02	78812.75	78974.62	79107.86	79265.76	79557.18	80213.68
	Height (%)	0.1	1.3	4.8	2.2	32.6	100.0	13.0

Supplementary Table 4: Primer sequences for PCR, qPCR, and sequence analysis of *ATP6AP1*

Region of ATP6AP1	Forward 5'->3'	Reverse 5'->3'
**Primers PCR**		
Exon 1	tgtaaaacgacggccagtATGCACTTGGTCCAATTACCTGCG	caggaaacagctatgaccAGCTGCAAGTCGCTGGTGATGTGGC
Exon 2	tgtaaaacgacggccagtTGCCGGTGTTTTTGTCGT	caggaaacagctatgaccTGGGACAAGGTCACTGAAGC
Exon 3	tgtaaaacgacggccagtACTTTGCTGGCCCTGAGAAT	caggaaacagctatgaccGGGATGGTTGACCCAGAAGA
Exon 4	tgtaaaacgacggccagtGGTATGGCTTGCCAGAGGA	caggaaacagctatgaccTTCTGGAAGGGCTGTGAGG
Exon 5	tgtaaaacgacggccagtGGGCACTGAGGTAAGAGTGT	caggaaacagctatgaccACTGCAGGCCAGCTCTCTCT
Exon 6	tgtaaaacgacggccagtAAGCAGACAGTGGGGAGTGTT	caggaaacagctatgaccCCACCTCCAAGCAGACTCAC
Exon 7	tgtaaaacgacggccagtCTGGCTGATGGGACTTTGAG	caggaaacagctatgaccATGACACCTCCTACATCCTCAC
Exon 8	tgtaaaacgacggccagtAAGGACCCAAGGCAGCTTAGGTAGG	caggaaacagctatgaccTACATGCCCAAGCCCAAG
Exon 9	tgtaaaacgacggccagtGTCAAGTGGGGACACTGCTT	caggaaacagctatgaccCAGCAAGAACTGGGACAGG
Exon 10	tgtaaaacgacggccagtTTGAGAGTCCTGTCCCTGC	caggaaacagctatgaccCACCGTCCCACCCTCAAC
**Primers sequence PCR**		
M13 tail	tgtaaaacgacggccagt	caggaaacagctatgacc
**Primers qPCR**		
ATP6AP1	TCAACCTGACTGGCTCCTTC	GAGGCGCTCCATGGTAAAC
ATP6AP1	CCCAGCATCTACTCCTTCCA	GGAGAACTGCTCCCCCATTA
PPIB	CGGAAAGACTGTTCCAAAAAC	GATTACACGATGGAATTTGCTG
GUSB	AGAGTGGTGCTGAGGATTGG	CCCTCATGCTCTAGCGTGTC

Supplementary Table 5: Plasmids used for Yeast complementation studies

Plasmid	Description	Reference
pRS316	Yeast shuttle vector, *CEN ARS URA3 Amp*^{r}	Sikorski and Hieter, 1989
pMR072	pRS316 *HA-VOA1*	Ryan et al. 2008
pMR0712	pRS316 *HA-voa1QQ*	Ryan et al. 2008
pMR092	pRS316 *HA-GFP-VOA1*	This study
pMR1312	pRS316 *HA-GFP-voa1QQ*	This study
pMR124	pRS316 *VOA1(1-25)-HA-VOA1(26-217)-ATP6AP1(421-470)-KKNN*	This study
pMR1210	pRS316 *VOA1(1-25)-HA-ATP6AP1(43-470)-KKNN*	This study
pMR1211	pRS316 *VOA1(1-25)-HA-ATP6AP1(43-470)*	This study
pMR1213	pRS316 *VOA1(1-25)-HA-ATP6AP1(251-470)-KKNN*	This study
pMR1213Y313C	pRS316 *VOA1(1-25)-HA-ATP6AP1(251-470)-KKNN* Y313C	This study
pMR1213E346K	pRS316 *VOA1(1-25)-HA-ATP6AP1(251-470)-KKNN* E346K	This study
pMR1213M428I	pRS316 *VOA1(1-25)-HA-ATP6AP1(251-470)-KKNN* M428I	This study
pMR1214	pRS316 *VOA1(1-25)-HA-ATP6AP1(251-470)*	This study
pMR1303	pRS316 *VOA1(1-25)-HA-GFP-ATP6AP1(251-470)-KKNN*	This study
pMR1303E346K	pRS316 *VOA1(1-25)-HA-GFP-ATP6AP1(251-470)-KKNN* E346K	This study
pMR1303M428I	pRS316 *VOA1(1-25)-HA-GFP-ATP6AP1(251-470)-KKNN* M428I	This study
pMR1304	pRS316 *VOA1(1-25)-HA-GFP-ATP6AP1(251-470)*	This study

Numbers in parentheses refer to amino acid residues

Supplementary References

[1] Sikorski, R. S. and P. Hieter (1989). "A system of shuttle vectors and yeast host strains designed for efficient manipulation of DNA in Saccharomyces cerevisiae." Genetics **122**(1): 19-27.

[2] Ryan, M., L. A. Graham, et al. (2008). "Voa1p functions in V-ATPase assembly in the yeast endoplasmic reticulum." Mol Biol Cell **19**(12): 5131-5142.
